# Taming the stability of Pd active phases through a compartmentalizing strategy toward nanostructured catalyst supports

**DOI:** 10.1038/s41467-019-09662-4

**Published:** 2019-04-08

**Authors:** Xinwei Yang, Qing Li, Erjun Lu, Zhiqiang Wang, Xueqing Gong, Zhiyang Yu, Yun Guo, Li Wang, Yanglong Guo, Wangcheng Zhan, Jinshui Zhang, Sheng Dai

**Affiliations:** 1 Key Laboratory for Advanced Materials and Research Institute of Industrial Catalysis, School of Chemistry and Molecular Engineering, East China University of Science and Technology, 130 Meilong Road, 200237 Shanghai, China; 2 State Key Laboratory of Photocatalysis on Energy and Environment College of Chemistry, Fuzhou University, 350116 Fuzhou, China; 3 Chemical Sciences Division Oak Ridge National Laboratory, University of Tennessee, Knoxville, TN 37996 USA; 4 Department of Chemistry, University of Tennessee, Knoxville, TN 37996 USA

## Abstract

The design and synthesis of robust sintering-resistant nanocatalysts for high-temperature oxidation reactions is ubiquitous in many industrial catalytic processes and still a big challenge in implementing nanostructured metal catalyst systems. Herein, we demonstrate a strategy for designing robust nanocatalysts through a sintering-resistant support via compartmentalization. Ultrafine palladium active phases can be highly dispersed and thermally stabilized by nanosheet-assembled γ-Al_2_O_3_ (NA-Al_2_O_3_) architectures. The NA-Al_2_O_3_ architectures with unique flowerlike morphologies not only efficiently suppress the lamellar aggregation and irreversible phase transformation of γ-Al_2_O_3_ nanosheets at elevated temperatures to avoid the sintering and encapsulation of metal phases, but also exhibit significant structural advantages for heterogeneous reactions, such as fast mass transport and easy access to active sites. This is a facile stabilization strategy that can be further extended to improve the thermal stability of other Al_2_O_3_-supported nanocatalysts for industrial catalytic applications, in particular for those involving high-temperature reactions.

## Abstract (web summary)

The design and synthesis of robust sintering-resistant nanocatalysts for high-temperature oxidation reactions remains challenging, even though the strategy of metal-support interactions has been extensively used. Here, the authors demonstrate an alternative strategy for designing robust nanocatalysts through a sintering-resistant support.

## Introduction

The development of thermally robust supported metal nanocatalysts that can undergo high-temperature oxidative or reductive processes is of great interest for industrial catalytic reactions, such as catalytic combustion of hydrocarbons at elevated temperatures and water-gas shift reactions under high temperatures and pressures^1–12^. However, owing to their low Tammann temperatures and high surface energies, catalytically active metal nanoparticles are thermodynamically unstable and tend to sinter or coalescence into larger particles during reactions, especially at high reaction temperatures^13–16^. In addition, phase transformations or the structural collapse of supports at elevated temperatures may exacerbate the sintering or encapsulation of metal nanoparticles^17–19^. Unfortunately, both such thermal behaviours will result in fast deactivation of the catalysts and thus hamper their practical applications in industry. To obtain a sintering-resistant supported nanocatalyst, it is essential to address these issues by employing a suitable host support that can strengthens the metal-support interaction to stabilize metal nanoparticles and provides good mechanical stability to maintain the structural integrity at elevated temperatures^20–30^.

**Fig. 1:**
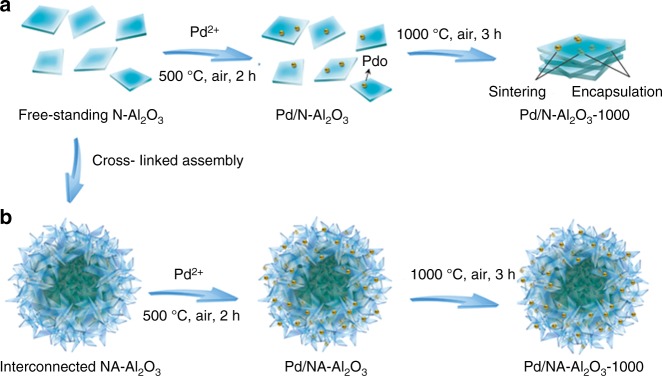
Illustration of stabilization of Pd active phases**. a** Pd active phases stabilized by N-Al_2_O_3_ in air at 1000 °C. **b** Pd active phases stabilized by NA-Al_2_O_3_ in air at 1000 °C

Gamma phase alumina (γ-Al_2_O_3_) is one of the most popular industrial catalysts and catalyst supports because of its compact crystal structure, excellent mechanical strength, high thermal stability, and robust chemical inertness^31–33^. Recently, it has been proved by high-resolution ^27^Al nuclear magnetic resonance (^27^Al NMR) that the coordinatively unsaturated pentacoordinate Al^3+^ (Al^3+^_penta_) centers present in γ-Al_2_O_3_ can act as binding sites for anchoring metals^34^. This finding is important in which it provides a facile approach to preparing thermally robust supported metal nanocatalysts^35,36^. To better utilize these Al^3+^_penta_ centers to stabilize metals, two-dimensional (2D) γ-Al_2_O_3_ nanosheets with open surface structures were synthesized^37–39^. However, because of their huge surface energies, these free-standing γ-Al_2_O_3_ nanosheets (N-Al_2_O_3_) tended to stack or agglomerate as bulk ones at elevated temperatures, or even under reaction^40^. This behavior not only induced the loss of the unique structural benefits of nanosheets for heterogeneous catalysis but also, more seriously, resulted in the sintering and/or encapsulation of metal nanoparticles, which rapidly deactivated the catalysts (Fig. 1). Therefore, finding a way to prevent the lamellar aggregation of nanosheets to preserve their unique electronic and structural advantages for stabilizing metals is of significant importance for the fabrication of thermally stable γ-Al_2_O_3_ nanosheet-supported catalysts.

The hierarchical assembly of 2D nanosheets into three-dimensional (3D) configurations can efficiently overcome the huge surface energies to prevent lamellar stacking, which is clearly demonstrated by the development of 3D graphene, flowerlike TiO_2_ flakes and nanospherical carbon nitride nanosheet frameworks, and other structures^41–48^. Inspired by this kind of structural arrangement, γ-Al_2_O_3_ nanosheets dominated by (110) facets are cross-linked as a hierarchical architecture with a unique flowerlike morphology (Fig. 1). For nanosheet-assembled Al_2_O_3_ (NA-Al_2_O_3_), the interconnected topology of the nanosheets provides enough structural rigidity against the surface energies to perfectly stabilize γ-Al_2_O_3_ nanosheets against lamellar aggregation and maintain the (110) facets at elevated temperatures. In addition, the thermally induced irreversible γ-to-α phase transformation is efficiently inhibited by spatially separating the nanosheets from each other to block the surface-controlled phase reactions (Supplementary Fig. 1). As a result, the NA-Al_2_O_3_ architecture serves as a robust host material with unique structural benefits to support metal nanoparticles, and the resultant nanocatalyst exhibits excellent thermal stability and hierarchical structural benefits for heterogeneous catalysis. For example, the Al^3+^_penta_ centers can serve as anchor sites to immobilize ultrafine metal nanoparticles on nanosheets with a strong metal-support interaction^34–36^. And the robust structural interconnectivities of the 3D architecture afford enough mechanical stability to protect the structural integrity against deformation and phase transformation at elevated temperatures, thus efficiently avoiding the sintering and encapsulation of metal nanoparticles caused by support material deformation^49–52^. Furthermore, the flowerlike morphology, with its multifaceted open surfaces, is favorable for mass transfer and made the active metal phases more easily accessible to the reacting molecules. Experimentally, both characteristics are beneficial for heterogeneous catalysis. Hence, it is significant that the ultrafine metals supported on the NA-Al_2_O_3_ architecture are thermally robust for high-temperature oxidative or reductive reactions.

Herein, NA-Al_2_O_3_ hierarchical architectures are fabricated and employed as the host materials to disperse and stabilize ultrafine palladium (Pd) active phases (Pd/NA-Al_2_O_3_) for high-temperature oxidative reactions. As a result of its robust thermal stability, Pd/NA-Al_2_O_3_ exhibits an excellent catalytic performance toward combustion of hydrocarbons (e.g. methane and propane) at elevated temperatures. Furthermore, this facile stabilization strategy can be further extended to improve the thermal stability of other Al_2_O_3_-supported nanocatalysts for industrial catalytic applications, in particular for those involving high-temperature reactions.

## Results

### Thermal behavior of NA-Al_2_O_3_ architectures

**Fig. 2:**
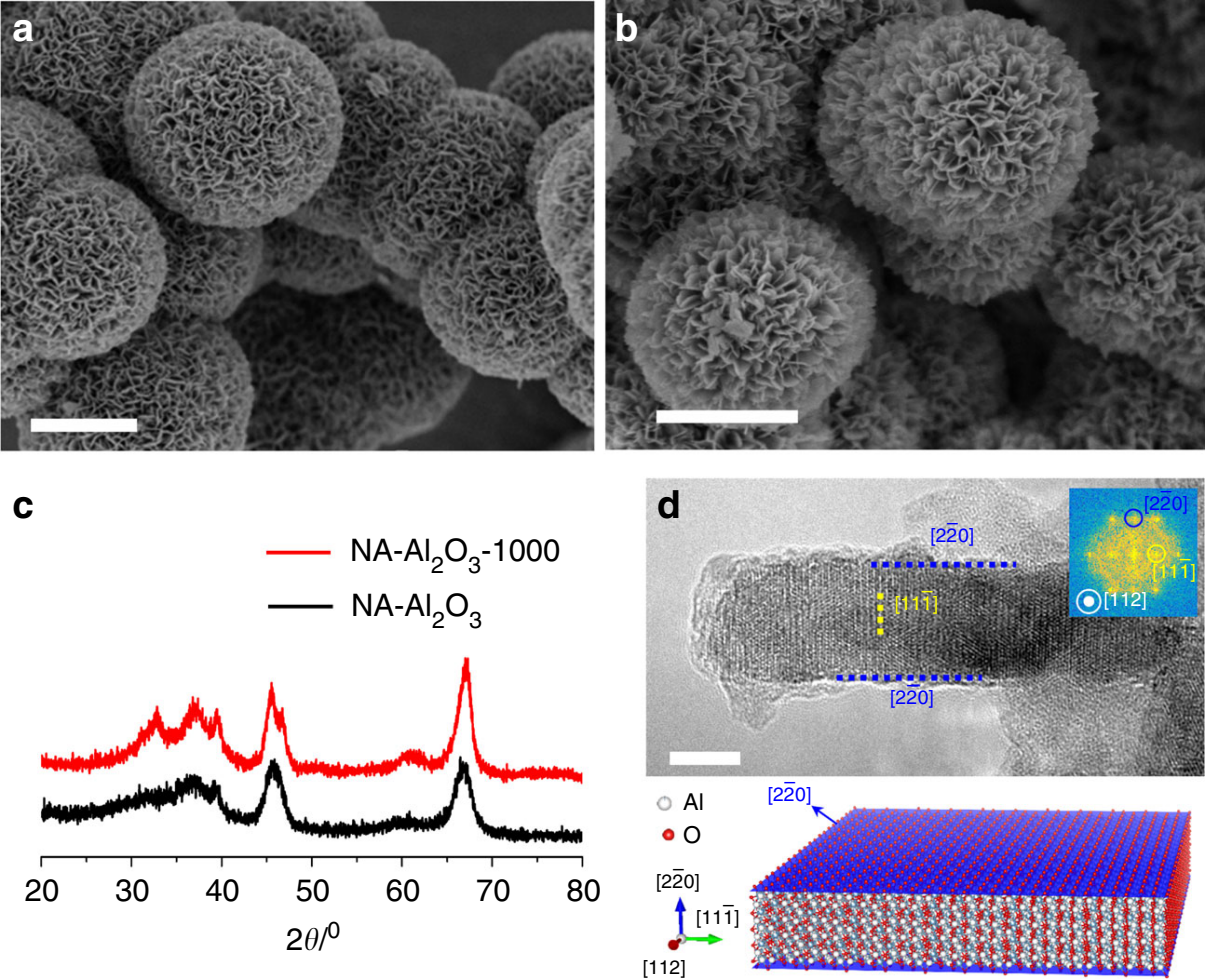
Thermal characterization of NA-Al_2_O_3_ host materials. **a** SEM images of NA-Al_2_O_3_. **b** SEM images of NA-Al_2_O_3_-1000. **c** XRD patterns of NA-Al_2_O_3_ and NA-Al_2_O_3_-1000. **d** HRTEM image of a typical nanosheet of NA-Al_2_O_3_-1000. Upper panel: It was aligned to an edge-on condition, namely, a condition that the basal surfaces (2$\overline 2$0) of nanosheets were in parallel with the electron beam. The inset is the FFT pattern recorded from the nanosheet. Lower panel: A schematic diagram showing the geometry of the nanosheets. The two basal planes of the nanosheet are colored blue. The scale bar in (**a** and **b** corresponds to 2 µm, and in **d** corresponds to 5 nm

Fig. 2 illustrates the thermal stability of NA-Al_2_O_3_. To evaluate the thermal behavior, the as-prepared NA-Al_2_O_3_ architectures were subjected to an annealing treatment at 1000 °C in air for 3 h (the resultant sample was denoted as NA-Al_2_O_3_-1000) and then were characterized by means of scanning electron microscopy (SEM), transmission electron microscopy (TEM), atomic force microscope (AFM), N_2_-sorption analysis, and X-ray diffraction (XRD). In Fig. 2a and Supplementary Fig. 2, the as-prepared NA-Al_2_O_3_ architectures were assembled from ~5-nm-thick flat nanosheets in a highly interconnected fashion to form a hollow spherical morphology; their diameters and shell thicknesses were determined to be 3–6 μm and 500–900 nm, respectively. Benefiting from the structural and mechanical advantages of such cross-linked nanosheet networks, which protected against lamellar aggregation, the hierarchical hollow architecture of NA-Al_2_O_3_ was quite robust and sustained 1000 °C annealing in air for 3 h without deformation (Fig. 2b and Supplementary Fig. 3). The specific surface area (S_BET_) of NA-Al_2_O_3_ and NA-Al_2_O_3_-1000 was determined to be 219 and 148 m^2^ g^−1^ (Supplementary Fig. 4), indicating that NA-Al_2_O_3_ has an excellent thermal stability comparable to commercial La-doped γ-Al_2_O_3_ (La-Al_2_O_3_, S_BET_ = 152 m^2^ g^−1^ for La-Al_2_O_3_, S_BET_ = 107 m^2^ g^−1^ for La-Al_2_O_3_-1000, Supplementary Fig. 5). In addition, the surface structure of the NA-Al_2_O_3_ also became much more open after the 1000 °C-annealing, which should be kinetically favorable for heterogeneous reactions. On the contrary, obvious structural deformation, particularly layer restacking/agglomeration occurred in the free-standing N-Al_2_O_3_ (Supplementary Fig. 6). It resulted in the evident decrease of S_BET_ from 198 to 78 m^2^ g^−1^, seriously counteracting the structural advantages of the nanosheet morphology for heterogeneous catalysis. The crystallinity of NA-Al_2_O_3_ was greatly improved by 1000 °C-annealing treatment, as indicated by the appearance of high-resolution XRD reflections at 32.8, 37.1, and 39.5, respectively, for (220), (311), and (222) γ-Al_2_O_3_ (Fig. 2c, JCPDS 10-0425). As a result of the spatial separation of the nanosheets from each other to block the surface-controlled phase reactions, the thermally induced phase transformation was well suppressed on the NA-Al_2_O_3_ architecture; whereas for N-Al_2_O_3_, an irreversible γ-to-θ phase transformation was observed after calcination in air at 1000 °C for 3 h (Fig. 2c vs. Supplementary Fig. 7). To further investigate the thermal stability of NA-Al_2_O_3_, its hierarchical architectures were deformed by mechanical grinding (denoted as NA-Al_2_O_3_-deformed) and then subjected to 1000 °C-annealing. Similar to the results obtained from N-Al_2_O_3_-1000, irreversible γ-to-θ phase transformation and significant decrease of S_BET_ were observed for NA-Al_2_O_3_-deformed-1000 (S_BET_ = 195 m^2^ g^−1^ for NA-Al_2_O_3_-deformed, S_BET_ _=_ 72 m^2^ g^−1^ for NA-Al_2_O_3_-deformed-1000, Supplementary Fig. 8), further demonstrating the important role of compartmentalization strategy in stabilizing 2D nanosheets from sintering and collapse. In Fig. 2d, the nanosheet was aligned to edge-on conditions, e.g. [112] zone axis of γ- Al_2_O_3_ to study the terminal surfaces of NA-Al_2_O_3_^53,54^. As indicated by the clearly resolved lattice fringes of (2$\overline 2$0) and (11$\overline 1$) planes, the preferred surfaces of NA-Al_2_O_3_ were (110) facets. A schematic diagram was also given to show the geometry of the nanosheet structure. Thus, we conclude that the NA-Al_2_O_3_ architecture with primarily exposed (110) facets is a thermally robust host material and can be used to prepare sintering-resistant supported nanocatalysts.

### Thermal behavior of Pd/NA-Al_2_O_3_ nanocatalysts

**Fig. 3:**
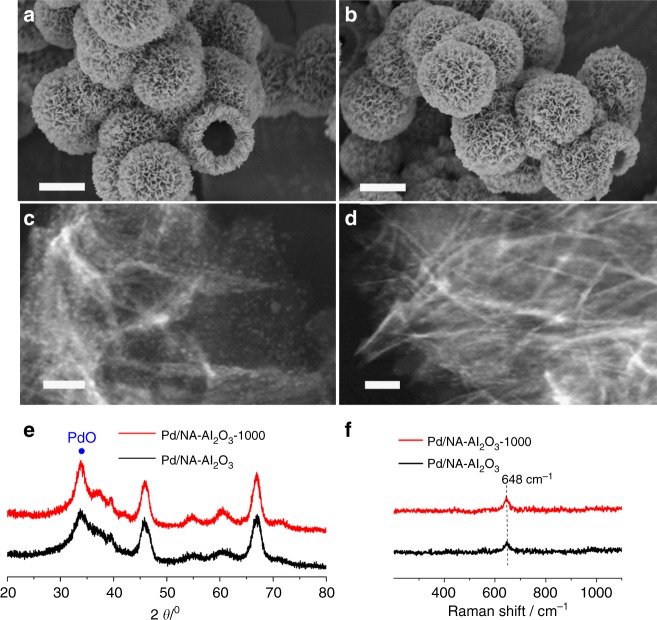
Thermal characterization of Pd/NA-Al_2_O_3_ nanocatalysts. **a** SEM images of Pd/NA-Al_2_O_3_. **b** SEM images of Pd/NA-Al_2_O_3_-1000. **c** TEM images of Pd/NA-Al_2_O_3_. **d** TEM images of Pd/NA-Al_2_O_3_-1000. **e** XRD patterns of Pd/NA-Al_2_O_3_-1000 and Pd/NA-Al_2_O_3_. **f** Raman spectra of Pd/NA-Al_2_O_3_-1000 and Pd/NA-Al_2_O_3_. The scale bar in **a** and **b** corresponds to 2 µm, and in **c** and **d** corresponds to 50 nm

The as-prepared NA-Al_2_O_3_ architectures were directly used to support Pd active phases through an incipient wet impregnation method to load Pd species. To better demonstrate the outstanding ability of NA-Al_2_O_3_ to stabilize metals, the metal loading was increased to 5 wt%, and the resultant sample was then subjected to annealing at 1000 °C in air for 3 h (the samples before and after 1000 °C-annealing are denoted Pd/NA-Al_2_O_3_ and Pd/NA-Al_2_O_3_-1000, respectively). Owing to the excellent stability of the NA-Al_2_O_3_ architecture (Fig. 2), Pd/NA-Al_2_O_3_ nanocatalysts were sufficiently robust to maintain their structural integrity under 1000 °C-annealing, thus efficiently avoiding the sintering and encapsulation of Pd nanoparticles caused by nanostructural deformation (Fig. 3a vs. 3b). The S_BET_ of Pd/NA-Al_2_O_3_ and Pd/NA-Al_2_O_3_-1000 was determined to be 152 and 145 m^2^ g^−1^, respectively (Supplementary Fig. 9). Such flowerlike hierarchical structure with well-preserved surface area was beneficial for Pd-catalyzed reactions because it facilitated the transport of reacting molecules to the surface of the catalyst to participate in the reaction^55,56^. The particle sizes of the Pd active phase were determined to be 2.6 ± 0.2 nm and 2.8 ± 0.2 nm for Pd/NA-Al_2_O_3_ and Pd/NA-Al_2_O_3_-1000, respectively (Fig. 3c, d and Supplementary Fig. 9). This result clearly confirms that the Pd active phase can be well stabilized by NA-Al_2_O_3_ against sintering at elevated temperatures. In sharp contrast, serious sintering of the Pd active phase with increasing the particle size from 2.5 ± 0.3 nm to 75.9 ± 17.7 nm and collapse of the Al_2_O_3_ nanosheets with decreasing the S_BET_ from 139 to 70 m^2^ g^−1^ occurred in Pd/N-Al_2_O_3_, owing to the poor thermal stability of the free-standing nanosheet structure (Supplementary Fig. 10). In addition, NA-Al_2_O_3_ with deformed nanostructure (Supplementary Fig. 8) was also used to support Pd species. In Supplementary Fig. 11, evident sintering of Pd active phases also happened to Pd/NA-Al_2_O_3_-deformed-1000, further demonstrating that the compartmentalization of nanosheets plays a critical role in stabilizing Pd active phases. To better study the influence of phase change of alumina on stabilizing Pd species, La-Al_2_O_3_ with an excellent thermal stability (Supplementary Fig. 5) was also employed for Pd loading. Similar to the results obtained from Pd/N-Al_2_O_3_-1000 and Pd/NA-Al_2_O_3_-deformed-1000, an obvious sintering behavior still happened to Pd/La-Al_2_O_3_-1000 (Supplementary Fig. 12). These three control experiments underline the structural advantages of nanosheet-assembly hierarchical architectures in stabilizing metals under elevated temperatures. In Supplementary Fig. 13, Pd/NA-Al_2_O_3_, Pd/N-Al_2_O_3_, and Pd/La-Al_2_O_3_ nanocatalysts were also subjected to an annealing treatment in wet air condition (10 vol% H_2_O) to evaluate their hydrothermal stability for practical catalytic applications. As expected, the ultrafine Pd active phases can be well still stabilized by NA-Al_2_O_3_ even after calcinated in wet air, whereas significant sintering of Pd phases occurred to N-Al_2_O_3_ and La-Al_2_O_3_ supported samples. This finding demonstrated that Pd/NA-Al_2_O_3_ is a promising nanocatalyst that can work under wet air conditions.

Figure 3e shows the crystal structure of Pd/NA-Al_2_O_3_ before and after thermal annealing. Using the unique structural benefits of the hierarchical architecture to suppress the thermally induced phase conversion confirmed that the γ phase was the predominant phase for the host NA-Al_2_O_3_ in both samples. The new diffraction peaks arising at 33.8 and 54.7° were identified as the characteristic (101) and (112) reflections of palladium oxide (PdO), and no other peak assigned to metallic Pd was observed^57,58^. This finding indicates that the Pd active phase was stabilized mainly as PdO nanoparticles on NA-Al_2_O_3_, even after harsh high-temperature treatment, rather than Pd nanoparticles. It was further confirmed by Raman spectra. In Fig. 3f, the formation of PdO on NA-Al_2_O_3_ is clearly identified by the appearance of a characteristic peak assigned to the B_1g_ mode of Pd-O at a Raman shift of 648 cm^−1^^59,60^. This finding is apparently inconsistent with the fact that PdO will transform into metallic Pd when being subjected to 1000 °C-annealing in air^61,62^. To better solve this issue, a control experiment that cooling down the Pd/NA-Al_2_O_3_ nanocatalyst in N_2_ was carried out after annealing the sample at 1000 °C in air. As demonstrated in Supplementary Fig. 14, the high-temperature induced decomposition of PdO indeed happened to Pd/NA-Al_2_O_3_ nanocatalyst, and the PdO was partially transformed into metallic Pd when cooled down in N_2_. But why is only PdO phase determined in Pd/NA-Al_2_O_3_-1000? The reason should be mainly attributed to that Pd active phases can be well stabilized in ultrasmall size by NA-Al_2_O_3_ (Supplementary Fig. 9), which facilitates the completely reoxidization of metallic Pd back to PdO phase by air during the cooling process. This finding was further confirmed by the results obtained from N-Al_2_O_3_, La-Al_2_O_3_, and NA-Al_2_O_3_-deformed supported samples. In Supplementary Fig. 15, owing to the evident sintering of Pd active phases, the arising of metallic Pd reflection at 40.1° was observed on Pd/N-Al_2_O_3_-1000, Pd/La-Al_2_O_3_-1000 and Pd/NA-Al_2_O_3_-deformed-1000 samples, indicating that PdO had been partially transformed into metallic Pd, even when they were cooled down in air. The Pd dispersion of Pd/NA-Al_2_O_3_, Pd/N-Al_2_O_3_ and Pd/La-Al_2_O_3_ before and after the 1000 °C-annealing treatment was summarized in Supplementary Table 1. Hence, it is clear that the Pd active phase was highly dispersed and stabilized as ultrasmall PdO nanoparticles by NA-Al_2_O_3_ at elevated temperatures in air, so supported Pd species in the oxidation state were favorable for high-temperature oxidation reactions. It should be noted that if PdO active phases had been previously reduced by H_2_, the resultant metallic Pd nanoparticles can be also well stabilized by NA-Al_2_O_3_ from sintering, even after 1000 °C-annealing in N_2_ (Supplementary Fig. 16a). In sharp comparison, sintering of metallic Pd was observed on N-Al_2_O_3_ and La-Al_2_O_3_ supported nanocatalysts (Supplementary Fig. 16b, c). Hence, by taking advantage of the unique nanostructure of NA-Al_2_O_3_, in particular compartmentalized 2D nanosheets with enlarged surface structure, Pd active species, either in oxidative or metallic phases can be well stabilized by NA-Al_2_O_3_ at elevated temperatures.

### Strong interaction between PdO and NA-Al_2_O_3_

**Fig. 4:**
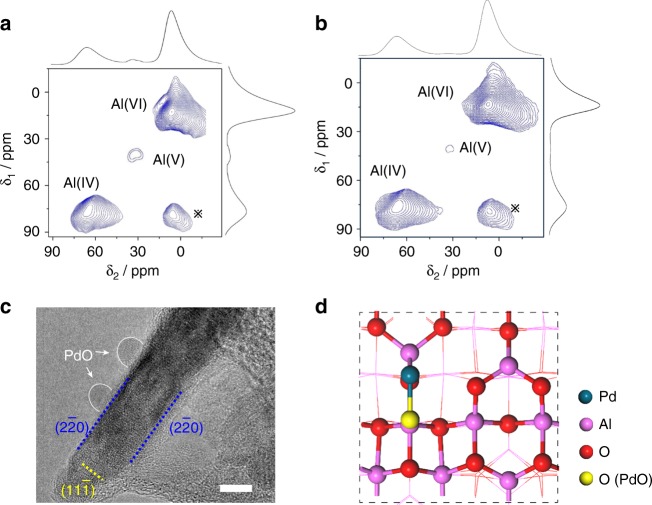
Structural characterization of NA-Al_2_O_3_ and Pd/NA-Al_2_O_3_. **a** Two-dimensional ^27^Al MQ MAS NMR spectra of NA-Al_2_O_3_. **b** Two-dimensional ^27^Al MQ MAS NMR spectra of Pd/NA-Al_2_O_3_. **c** TEM image of Pd/NA-Al_2_O_3_-1000. **d** Calculated structure of Pd/NA-Al_2_O_3_. The scale bar in **c** corresponds to 5 nm

In Fig. 4, the interaction between PdO and NA-Al_2_O_3_ is investigated by 2D ^27^Al NMR spectra, high-resolution (HR)-TEM imaging, and theoretical calculation. In the NMR spectra, three distinct peaks centered at 7, 32, and 65 ppm chemical shifts are observed on the as-prepared NA-Al_2_O_3_ host materials (Fig. 4a), which were assigned to the Al^3+^ ions in octahedral (Al^3+^_octa_), pentahedral (Al^3+^_penta_) and tetrahedral (Al^3+^_tetra_) coordination, respectively^63–65^. Among them, the Al^3+^_penta_ sites are considered as the primary anchoring sites for active catalytic phases and should play an essential role in the dispersion and stabilization of Pd oxide species^34–36^. Upon loading of the PdO on NA-Al_2_O_3_, the intensity of the peak Al^3+^_penta_ decreased significantly, indicating that most of the Al^3+^_penta_ sites had been used to immobilize Pd oxides on Al_2_O_3_ nanosheets (Fig. 4b)^37^. In Fig. 4c, the growth of PdO onto Al_2_O_3_ is confirmed by the HRTEM images, where Pd active phases sitting on the (110) surfaces of γ-Al_2_O_3_ nanosheets was clearly resolved. The energetics of the interaction of PdO with the (110) facets of γ-Al_2_O_3_ were studied by theoretical calculations In Fig. 4d, the sitting of PdO on (110) facets of γ-Al_2_O_3_ is exothermic, and the corresponding adsorption energy is determined to be 3.16 eV. This finding suggested that the Pd active phase supported on the (110) facets of γ-Al_2_O_3_ is thermodynamically stable, and large energy would be required for Pd-O bond breaking if the Pd phase started to migrate and sinter. Hence, the (110) facets of γ-Al_2_O_3_ thermodynamically facilitated the stabilization of the Pd active phase against sintering. The preservation of the structural integrity of γ-Al_2_O_3_ nanosheets to retain their (110) facets is therefore favorable for the preparation of robust sintering-resistant catalysts.

### Catalytic performance in methane combustion

**Fig. 5:**
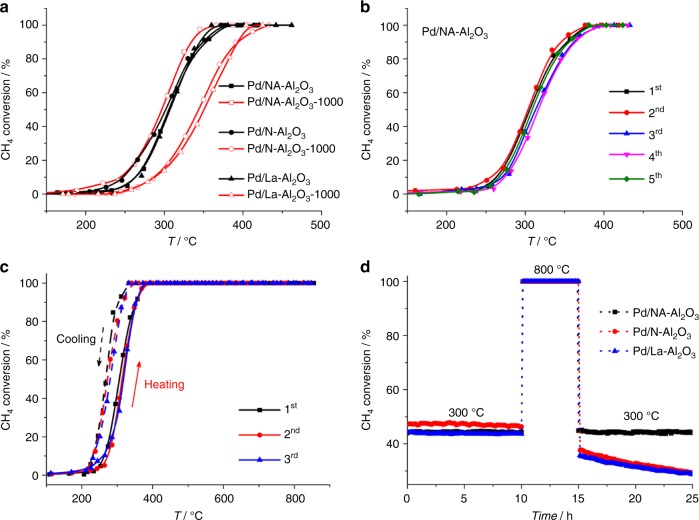
Catalytic combustion of methane on Pd/Al_2_O_3_ nanocatalysts. **a** CH_4_ conversion vs reactor temperature. **b** Repeating ignition−extinction cycles of methane conversion on Pd/NA-Al_2_O_3_. **c** Light-off curves of methane conversion on Pd/NA-Al_2_O_3_ at temperatures between 100–850 °C. **d** Long-term combustion of methane at 300-800-300 °C on Pd/NA-Al_2_O_3_, Pd/N-Al_2_O_3_ and Pd/La-Al_2_O_3_

Owing to the unique catalytic ability of Pd species in oxidation reactions, synthesized Pd/Al_2_O_3_ catalysts were subjected to methane oxidation at elevated temperatures for the purpose of better evaluating their catalytic performance, particularly thermal stability and catalytic durability. In Fig. 5a, obvious size-dependent catalytic behavior toward methane oxidation was observed on Pd/Al_2_O_3_ nanocatalysts. For example, a significant loss of activity was determined for Pd/N-Al_2_O_3_ and Pd/La-Al_2_O_3_ after 1000 °C-annealing, which was due to the evident growth and/or encapsulation of PdO under high temperatures (Pd/N-Al_2_O_3_ vs. Pd/N-Al_2_O_3_-1000, and Pd/La-Al_2_O_3_ vs. Pd/La-Al_2_O_3_-1000). In contrast, the catalytic activity of Pd/NA-Al_2_O_3_ was actually slightly better after thermal annealing (Pd/NA-Al_2_O_3_ vs. Pd/NA-Al_2_O_3_-1000). According to the literature, this improved performance is attributable to the good stabilization of PdO nanoparticles without sintering and the strengthening of the metal-support interaction by high-temperature annealing^66–69^. In Supplementary Fig. 17, a similar size-dependent catalytic behavior toward propane combustion was also observed, further confirming the thermal-induced size evolution of PdO nanoparticles in these nanocatalysts. To evaluate their catalytic stability, several control experiments were carried out. First are the successive ignition-extinction cycles for methane oxidation. For Pd/NA-Al_2_O_3_, the catalytic performance was rather robust without activity loss over five runs (Fig. 5b); whereas for Pd/N-Al_2_O_3_ and Pd/La-Al_2_O_3_, a significant loss of activity toward to a higher reaction temperature occurred immediately in the second run (Supplementary Fig. 18), due to the sintering of PdO nanoparticles at the first run. To indicate the stability under hydrothermal condition, water vapor (~10 vol%) was introduced to the reaction system. In Supplementary Fig. 19, Pd/NA-Al_2_O_3_ still exhibited a robust catalytic durability over Pd/N-Al_2_O_3_ and Pd/La-Al_2_O_3_ nanocatalysts for the catalytic combustion of methane in the present of water (~10 vol%), because of its sintering-resistant PdO nanoparticles in wet air (Supplementary Fig. 13). To demonstrate that PdO phases are stabilized during methane combustion, the heating/cooling ramps were collected. In Fig. 5c and Supplementary Fig. 20, the typical hysteresis curve caused by PdO-Pd-PdO transformation during cooling process was absent in all Pd/Al_2_O_3_ nanocatalysts, indicating that Pd active phases had been well stabilized in oxidative state for methane oxidation^70^. As a result of being heated to a higher temperature (850 vs. 450 °C) to induce obvious sintering, the activity loss of Pd/N-Al_2_O_3_ and Pd/La-Al_2_O_3_ became much more evidence in the light-off curves (Fig. 5a vs Supplementary Fig. 20). To better study the influence of reaction temperatures on catalytic activity, long-term operation of methane combustion at alternating reaction temperatures (e.g. 300-800-300 °C) was carried out. In Fig. 5d, each of the three catalysts exhibited a comparable catalytic stability in the first 10 h-run at 300 °C; but after being accelerated aging at 800 °C for 5 h, a significant loss of activity was observed for Pd/N-Al_2_O_3_ and Pd/La-Al_2_O_3_ when ramping the reaction temperature back to 300 °C. Interesting, Pd/NA-Al_2_O_3_ demonstrated a robust catalytic durability during this process. Hence, it can be concluded that the nanosheet-interconnected hierarchical architecture played an essential role in stabilizing PdO nanoparticles for catalysis, and the resultant Pd/NA-Al_2_O_3_ was a robust sintering-resistant catalyst suitable for high-temperature oxidation reactions (Supplementary Fig. 21).

## Discussion

In this study, we demonstrated that ultrafine Pd active phases were well stabilized by an NA-Al_2_O_3_ architecture as a promising sintering-resistant nanocatalyst for high-temperature oxidation reactions. Such cross-linked γ-Al_2_O_3_ nanosheets with flowerlike morphologies provided enough structural rigidity to efficiently suppress lamellar aggregation and the irreversible phase transformation of γ-Al_2_O_3_ nanosheets at elevated temperatures to avoid the sintering and encapsulation of Pd active phases. In addition, the flowerlike morphologies with multifaceted open surfaces demonstrated significant structural advantages for heterogeneous reactions, such as fast mass transport and easily accessible active sits. As a result, Pd/NA-Al_2_O_3_ nanocatalysts exhibited excellent catalytic activity and durability for methane combustion at high temperatures. We hope this facile synthetic strategy can be further extended to improve the thermal stability of other Al_2_O_3_-supported nanocatalysts for industrial catalytic applications, particularly those involving high-temperature reactions.

## Methods

### Synthesis of nanosheet-assembled Al_2_O_3_ (NA-Al_2_O_3_)

In all, 1.51 g of Al(NO_3_)_3_·9H_2_O, 0.70 g of K_2_SO_4_, and 0.50 g of CO(NH_2_)_2_ were dissolved in 80 mL deionized water. Then the obtained mixture was transferred into a 100 mL Teflon-lined stainless steel autoclave and heated at 180 °C for 3 h. When cooled to room temperature, the white precipitate was collected by filtration, dried at 80 °C for 12 h and finally calcined at 500 °C for 2 h.

### Synthesis of Al_2_O_3_ nanosheet (N-Al_2_O_3_)

The typical procedure to synthesize thin alumina nanosheet was as follows: 0.60 g of Al(NO_3_)_3_·9H_2_O, 0.24 g of lysine, and 0.77 g of CO(NH_2_)_2_ were dissolved in 80 mL deionized water to form a homogeneous solution under magnetic stirring. Then the solution was transferred into a 100 mL Teflon-lined stainless autoclave and heated at 100 °C for 24 h. After cooled to room temperature, the white precipitate was filtered, washed with deionized water and anhydrous alcohol several times, and then dried at 80 °C for 12 h. The sample was gained after calcination of the powder in air at 500 °C for 2 h with a heating rate of 1 °C min^−1^.

### Preparation of Pd/NA-Al_2_O_3_

5 wt% Pd active phases were loaded on NA-Al_2_O_3_ by incipient wet impregnation method and dried at 80 °C for 12 h. The obtained samples were then calcined at 500 °C for 2 h. Pd/N-Al_2_O_3_ and Pd/La-Al_2_O_3_ were prepared with the same method.

### Preparation of Pd/NA-Al_2_O_3_-1000

The as-prepared Pd/NA-Al_2_O_3_, Pd/N-Al_2_O_3_, and Pd/La-Al_2_O_3_ were calcined at 1000 °C for 3 h. Pd/N-Al_2_O_3_-1000 and Pd/La-Al_2_O_3_-1000 were prepared with the same method.

### Characterization

The powder X-ray diffraction (XRD) patterns were collected on a Bruker D8 Focus diffractometer with Cu Kα radiation (*λ* = 1.54056 Ǻ, operated at 40 kV and 40 mA). The X-ray photoelectron spectroscopy (XPS) were obtained at 25 °C on a PHI-Quantera SXM spectrometer with Al Kα (1486.6 eV) radiation as the excitation source at ultra-high vacuum (6.7 × 10^−8^ Pa). All binding energies (BE) were determined with respect to the C1s line (284.8 eV) originating from adventitious carbon. The morphologies of samples were investigated by the field emission scanning electron microscopy (FE-SEM) images obtained by a NOVA NanoSEM 450 instrument operated at the beam energy of 5 kV. High-angle annular dark-field (HAADF) scanning transmission electron microscopy (STEM) images and high resolution TEM images were obtained on a JEM-ARM200F TEM/STEM with a guaranteed resolution of 0.08 nm. Before microscopy examination, the catalyst powders were ultrasonically dispersed in ethanol and then a drop of the solution was put onto a copper grid coated with a thin lacey carbon film. Raman spectra were recorded on a Renishaw Raman spectrometer under ambient conditions, and the 514 nm line of a Spectra Physics Ar^+^ laser was used for an excitation. The laser beam intensity was 2 mW, and the spectrum slit width was 3.5 cm^−1^. Solid state ^27^Al MAS NMR was performed at room temperature on a Bruker AVANCE III 500 MHz solid-state NMR spectrometer, operating at a magnetic field of 11.7 T. The corresponding ^27^Al Larmor frequency was 130.28 MHz. All spectra were recorded at a sample spinning rate of 4 kHz. Each spectrum was acquired using a total of 2000 scans with a recycle delay time of 0.5 s and an acquisition time of 0.018 s. All spectra were externally referenced (i.e., the 0 ppm position) to an 1 M Al(NO_3_)_3_ aqueous solution. The Pd dispersion was measured by CO chemisorption method on the Autochem 2920 II apparatus. Before the test, the sample was pretreated in a flow (40 mL min^−1^) of 20 vol% O_2_ balanced with Ar at 500 °C for 1 h. Then the sample was first reduced in a flow (40 mL min^−1^) of 5 vol% H_2_ balanced with N_2_ at 200 °C for 1 h. After cooled to room temperature in a flow of He (40 mL min^−1^), several pulses of CO (1 vol% CO balanced with He) were introduced into the sample until no more adsorption was observed. The stoichiometry for Pd: CO was taken be unity.

### Computational method and models

Density functional theory (DFT) calculations were carried out using the Vienna Ab-initio Simulation Package (VASP). The spin-polarized projector augmented wave (PAW) method and the Perdew-Burke-Ernzerhof (PBE) electron exchange-correlation functional of the generalized gradient approximation (GGA) were applied in our calculations. The kinetic energy cut-off for the wave function expanded in the plane-wave basis was set as 400 eV. To optimize the structures, the calculation was performed until the maximum force upon each relaxed atom was less than 0.05 eV Å^−1^. The vacuum height was set as 10 Å to eliminate the interaction between neighboring slabs. The adsorption energy (*E*_ads_) was calculated as followed Eq. (1):

1 $$E_{\mathrm{ads}}{\mathrm{ = }} - \left( {E_{\mathrm{substrate}}{\mathrm{ + }}E_{\mathrm{PdO}} - E_{\mathrm{total}}} \right)$$

where *E*_total_ is the calculated total energy of the adsorption PdO cluster, *E*_substrate_ is the energy of the clean substrate and *E*_PdO_ is the energy of optimized PdO cluster in the vacuum.

In order to study the adsorption of PdO cluster on γ-Al_2_O_3_(110) surfaces, we used the following model: a 1 × 2 surface cell was used to construct a four-atomic-layer Al_2_O_3_(110) slab, and the top three layers of the Al_2_O_3_(110) slab were allowed to relax. The Brillouin-zone integration was performed along with a 2 × 2 × 1 Monkhorst-Pack grid for the (110) surface slabs.

### Catalytic combustion of methane and propane

The catalytic activity of the catalyst for CH_4_ combustion was evaluated in a fixed-bed reactor containing 200 mg of catalyst at atmospheric pressure, and the feed gas consisted of 1 vol% CH_4_, 20 vol% O_2_, 10 vol% H_2_O (when used) and Ar. The total gas flow rate was 50 mL min^−1^, and the corresponding gas hourly space velocity (GHSV) was 15 000 mL h^−1^ g_cat_^−1^. The inlet and outlet CH_4_ concentration were measured by Agilent GC 7890 A. The CH_4_ conversion (X_CH4_) was calculated using the following Eq. (2):

2 $${\mathrm{X}}_{{\mathrm{CH}}_4}{\mathrm{\% }} = \frac{{\left[ {{\mathrm{CH}}_4} \right]_{{\mathrm{in}}} - \left[ {{\mathrm{CH}}_4} \right]_{{\mathrm{out}}}}}{{\left[ {{\mathrm{CH}}_4} \right]_{{\mathrm{in}}}}} \times 100$$

where [CH_4_]_in_ and [CH_4_]_out_ are the CH_4_ concentrations in the inlet and outlet gas, respectively.

The catalytic activity of the catalyst for C_3_H_8_ combustion was evaluated in a fixed-bed reactor containing 100 mg of catalyst at atmospheric pressure, and the feed gas consisted of 0.2 vol% C_3_H_8_, 2 vol% O_2_, and Ar. The total gas flow rate was 50 mL/min, and the corresponding GHSV was 30 000 mL h^−1^ g_cat_^−1^. The inlet and outlet C_3_H_8_ concentration were measured by by an online gas chromatograph (GC-2060) that was equipped with an FID. The C_3_H_8_ conversion (X_C3H8_) was calculated using the following Eq. (3):

3 $${\mathrm{X}}_{{\mathrm{C}}_3{\mathrm{H}}_8}{\mathrm{\% }} = \frac{{\left[ {{\mathrm{C}}_3{\mathrm{H}}_8} \right]_{{\mathrm{in}}} - \left[ {{\mathrm{C}}_3{\mathrm{H}}_8} \right]_{{\mathrm{out}}}}}{{[{\mathrm{C}}_3{\mathrm{H}}_8]_{{\mathrm{in}}}}} \times 100$$

where [C_3_H_8_]_in_ and [C_3_H_8_]_out_ are the C_3_H_8_ concentrations in the inlet and outlet gas, respectively.

## Supplementary information

Supplementary information

Peer Review

## Data Availability

All relevant data are available from the authors on reasonable request.
