# Supplementary material for: Taming the stability of Pd active phases through a compartmentalizing strategy toward nanostructured catalyst supports
Source: Nat Commun. 2019 Apr 8;10:1611. doi: 10.1038/s41467-019-09662-4 (PMC6453908; doi:10.1038/s41467-019-09662-4)
Supplement: Supplementary file 1 — Supplementary information [file 41467_2019_9662_MOESM1_ESM.pdf]

Supplementary information

Taming the stability of Pd active phases through a compartmentalizing strategy toward nanostructured catalyst supports

Yang et al.

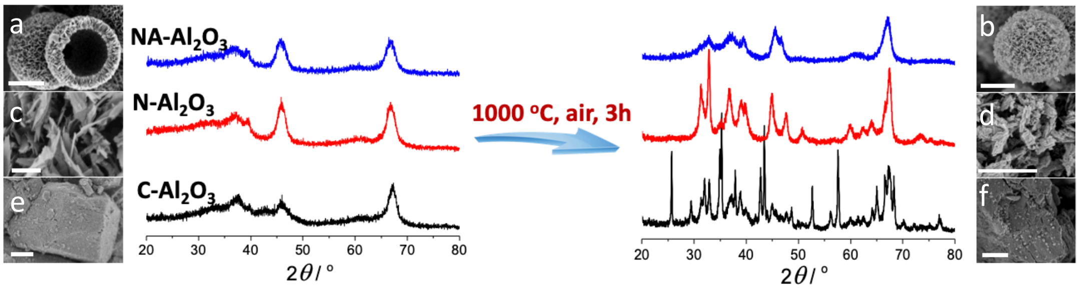

Supplementary Figure 1. SEM images and XRD patterns of NA-Al_2O_3, N-Al_2O_3 and C-Al_2O_3 before and after 1000 °C-annealing. The scale bar in (**a**), (**b**) and (**e**) corresponds to 2 μm, in (**c**) corresponds to 500 nm, in (**d**) corresponds to 1 μm, and in (**f**) corresponds to 5 μm.

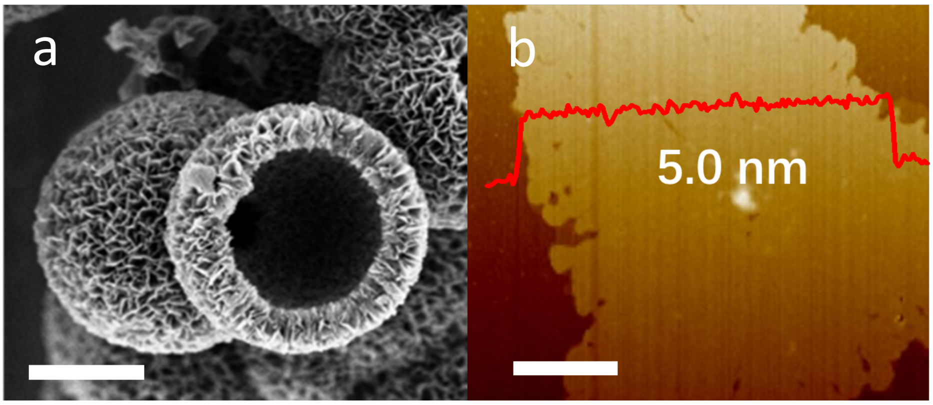

Supplementary Figure 2. SEM (**a**) and AFM (**b**) images of NA-Al_2O_3. The scale bar in (**a**) and (**b**) corresponds to 2 μm and 200 nm, respectively.

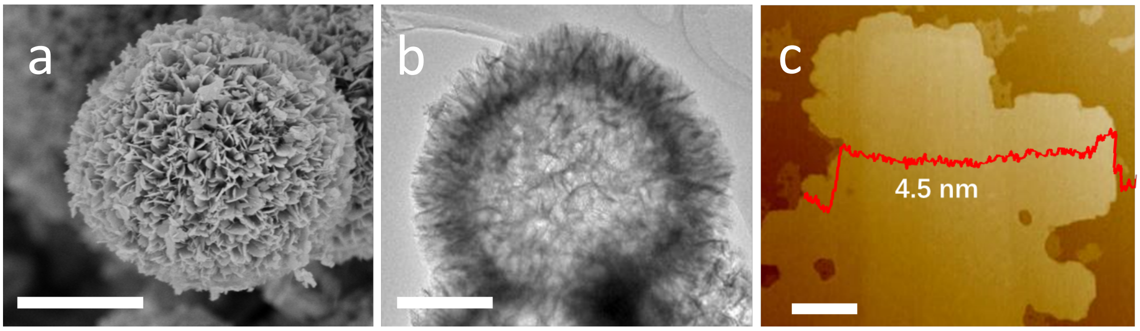

Supplementary Figure 3. SEM (**a**), TEM (**b**) and AFM (**c**) images of NA-Al_2O_3-1000. The scale bar in (**a**), (**b**) and (**c**) corresponds to 2 µm, 1 µm and 200 nm, respectively.

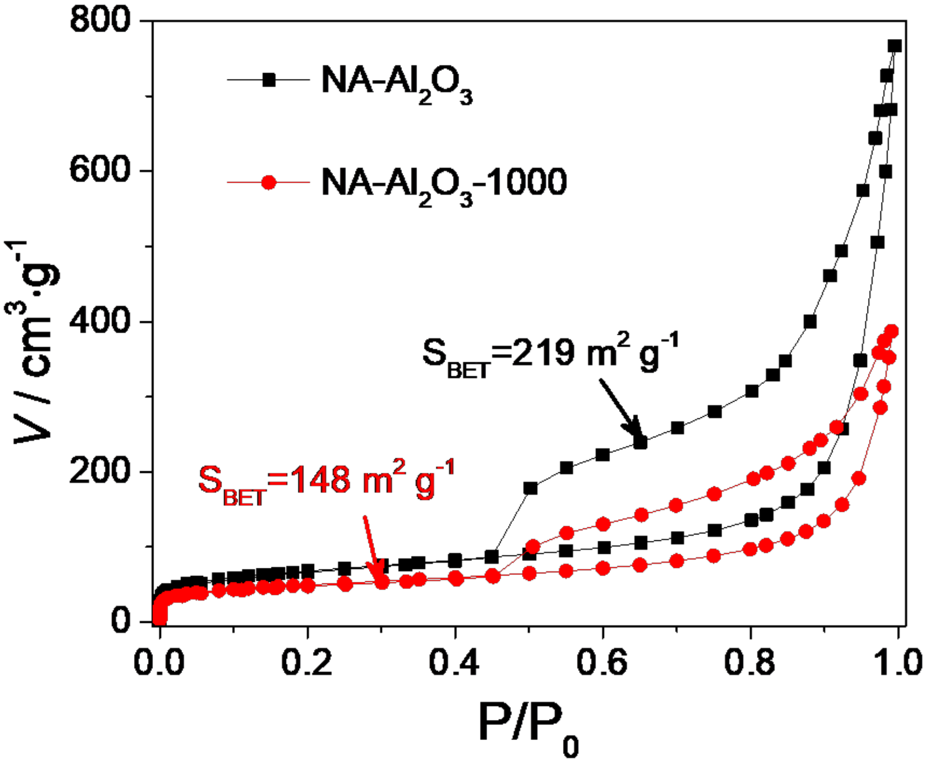

Supplementary Figure 4. N_2-sorption isotherms of NA-Al_2O_3 and NA-Al_2O_3-1000

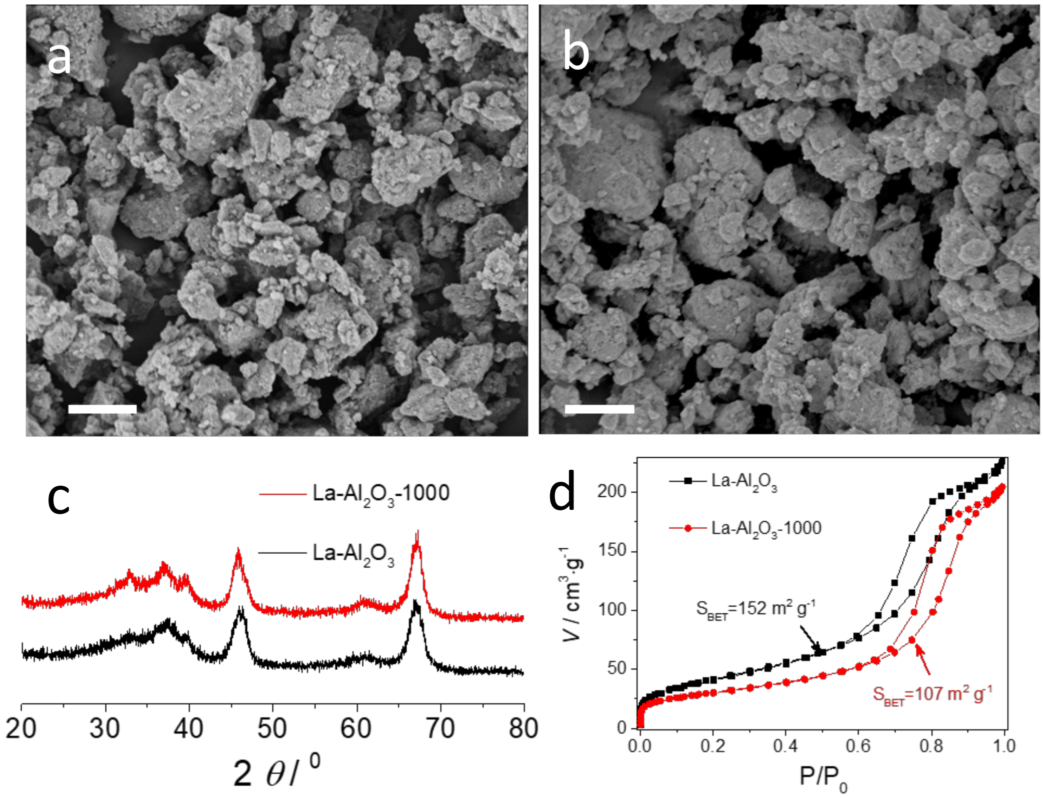

Supplementary Figure 5. a SEM images of La-Al_2O_3. **b** SEM images of La-Al_2O_3-1000. **c** XRD patterns of of La-Al_2O_3 and La-Al_2O_3-1000. **d** N_2-sorption isotherms of La-Al_2O_3 and La-Al_2O_3-1000. The scale bar in (**a**) and (**b**) corresponds to 2 μm.

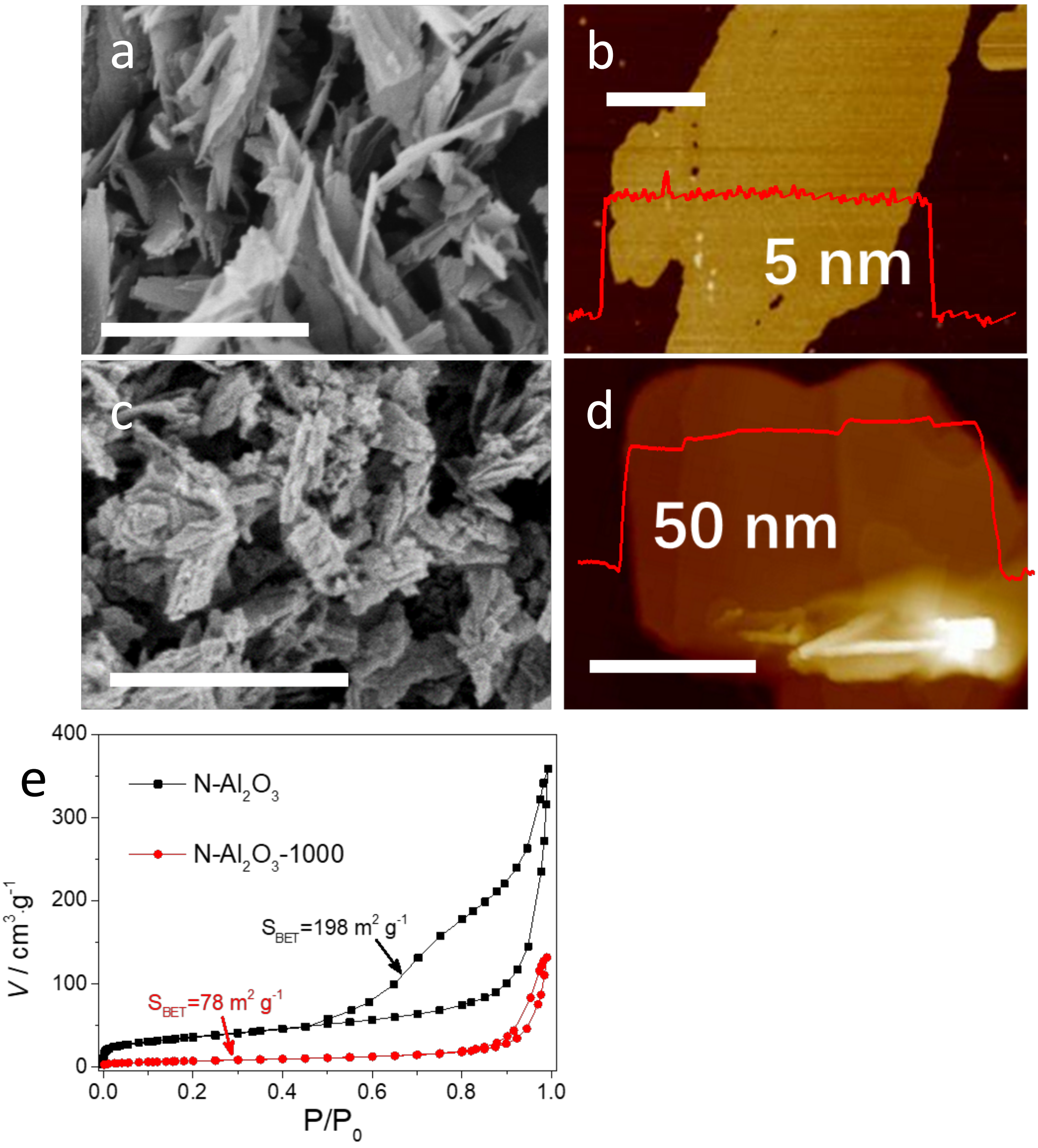

Supplementary Figure 6. SEM (**a**) and AFM (**b**) images of N-Al_2O_3. SEM (**c**) and AFM (**d**) images of N-Al_2O_3-1000. **e** N_2-sorption isotherms of N-Al_2O_3 and N-Al_2O_3-1000. The scale bar in (**a**) and (**c**) corresponds to 1 μm, in (**b**) and (**d**) corresponds to 500 nm.

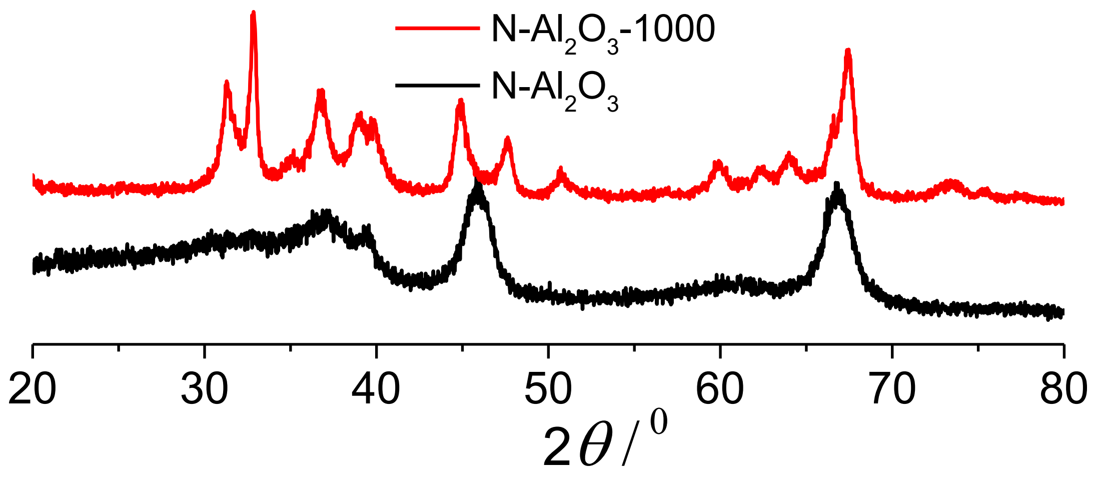

Supplementary Figure 7. XRD patterns of N-Al_2O_3 before and after 1000 °C-annealing.

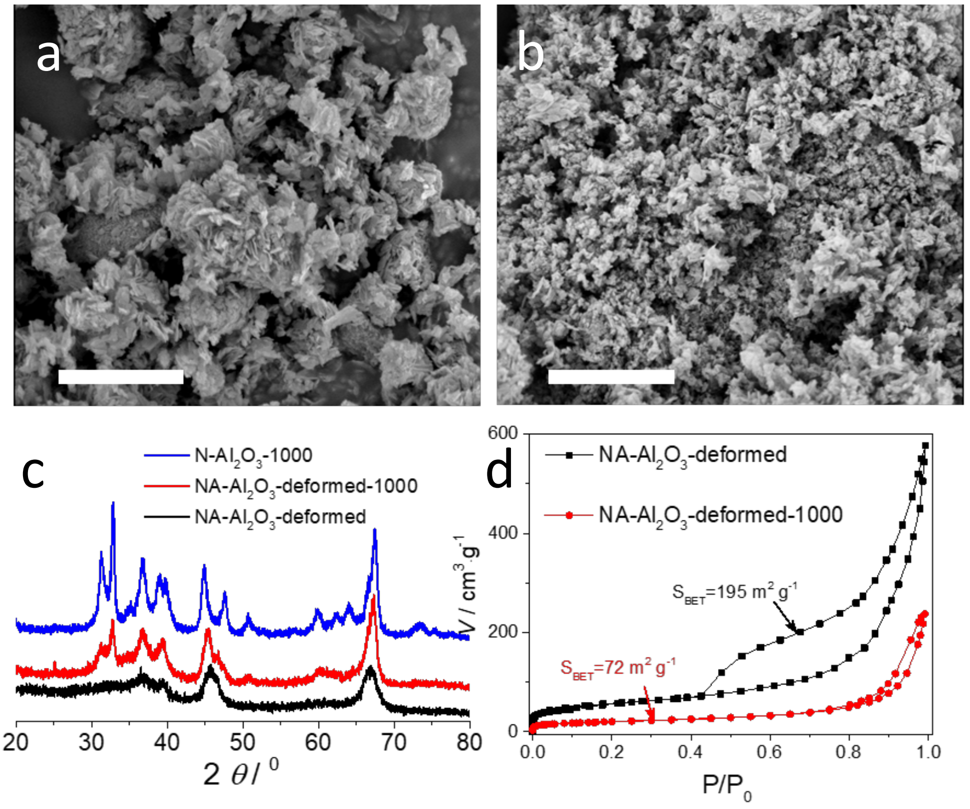

Supplementary Figure 8. a SEM images of NA-Al_2O_3-deformed. **b** SEM images of NA-Al_2O_3-deformed-1000. **c** XRD patterns of of NA-Al_2O_3-deformed and NA-Al_2O_3-deformed-1000. **d** N_2-sorption isotherms of NA-Al_2O_3-deformed and NA-Al_2O_3-deformed-1000. The scale bar in (**a**) and (**b**) corresponds to 2 μm.

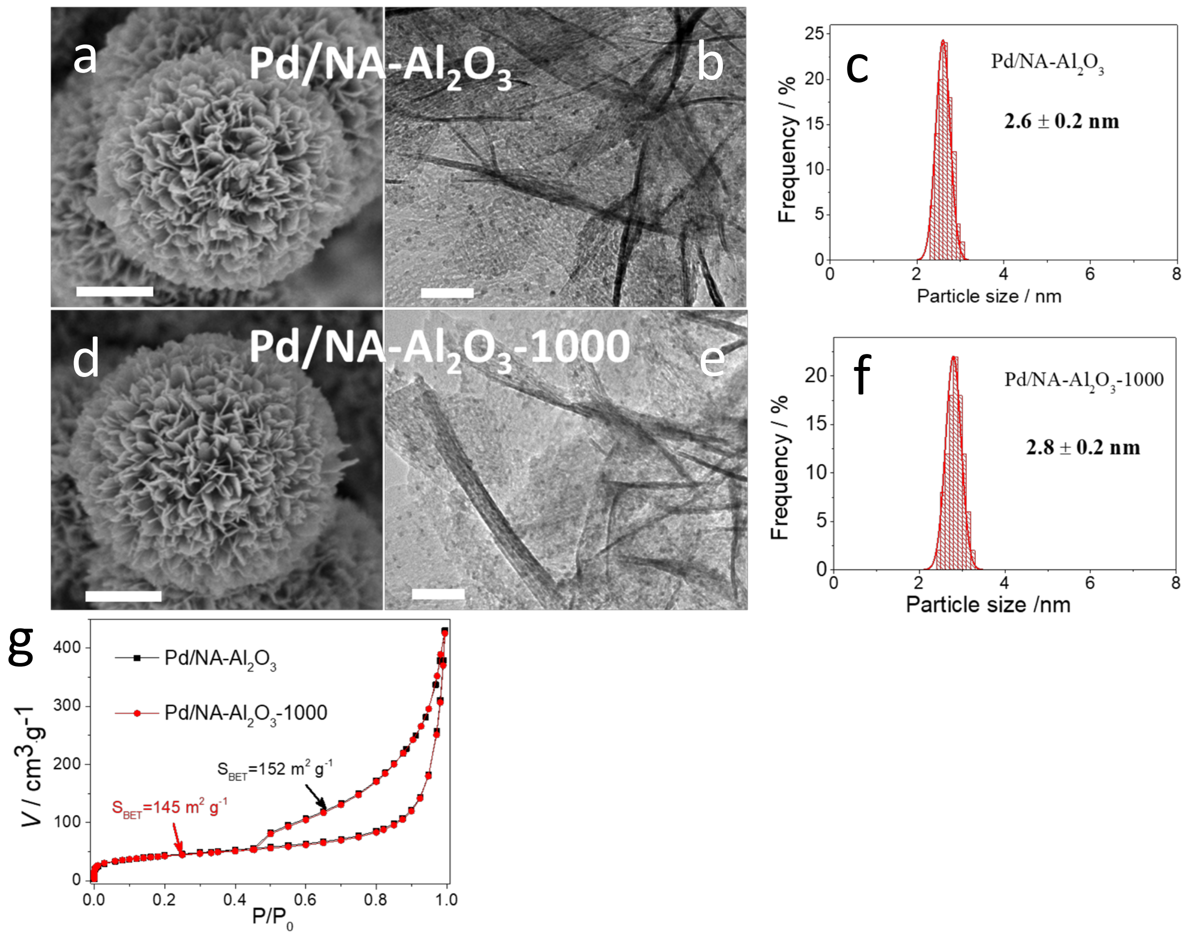

Supplementary Figure 9. a SEM images of Pd/NA-Al_2O_3. **b** TEM images of Pd/NA-Al_2O_3. **c** Particle-size distribution of Pd/NA-Al_2O_3. **d** SEM images of Pd/NA-Al_2O_3-1000. **e** TEM images of Pd/NA-Al_2O_3-1000. **f** Particle-size distribution of Pd/NA-Al_2O_3-1000. **g** N_2-sorption isotherms Pd/NA-Al_2O_3 and Pd/NA-Al_2O_3-1000. The scale bar in (**a**), (**b**), (**d**) and (**e**) corresponds to 1 μm, 50 nm, 1 μm and 50 nm, respectively.

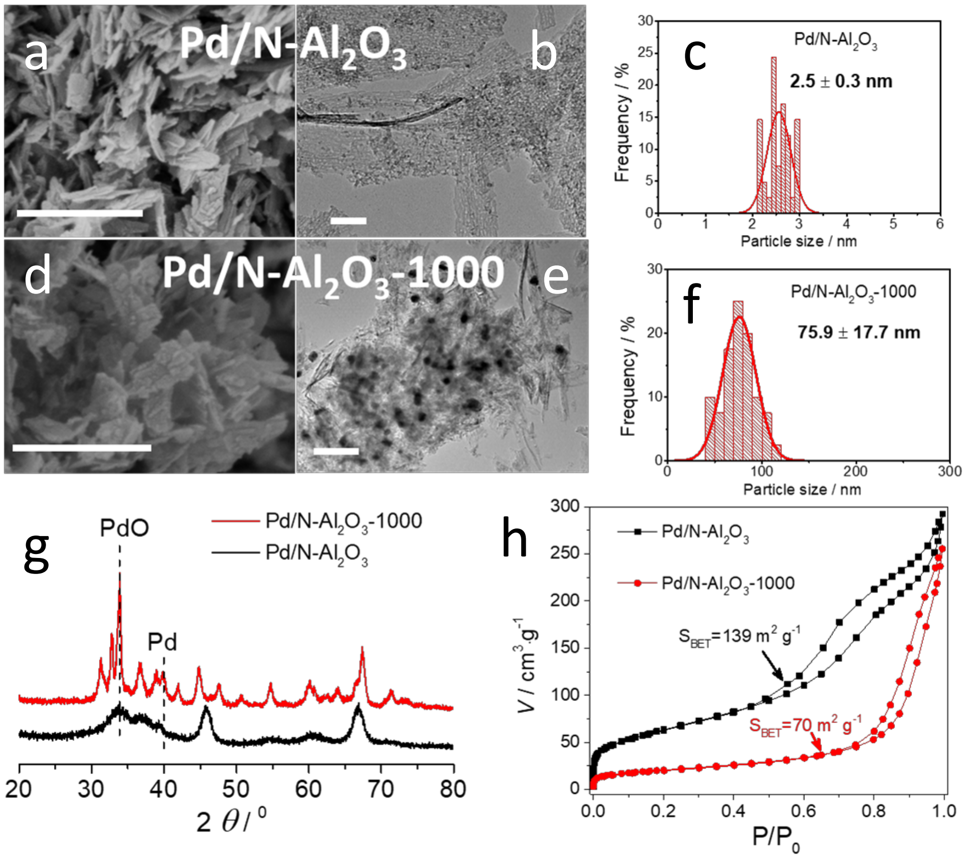

Supplementary Figure 10. a SEM images of Pd/N-Al_2O_3. **b** TEM images of Pd/N-Al_2O_3. **c** Particle-size distribution of Pd/N-Al_2O_3. **d** SEM images of Pd/N-Al_2O_3-1000. **e** TEM images of Pd/N-Al_2O_3-1000. **f** Particle-size distribution of Pd/N-Al_2O_3-1000. **g** XRD patterns of Pd/N-Al_2O_3 and Pd/N-Al_2O_3-1000. **h** N_2-sorption isotherms of Pd/N-Al_2O_3 and Pd/N-Al_2O_3-1000. The scale bar in (**a**), (**b**), (**d**) and (**e**) corresponds to 1 μm, 50 nm, 1 μm and 500 nm, respectively.

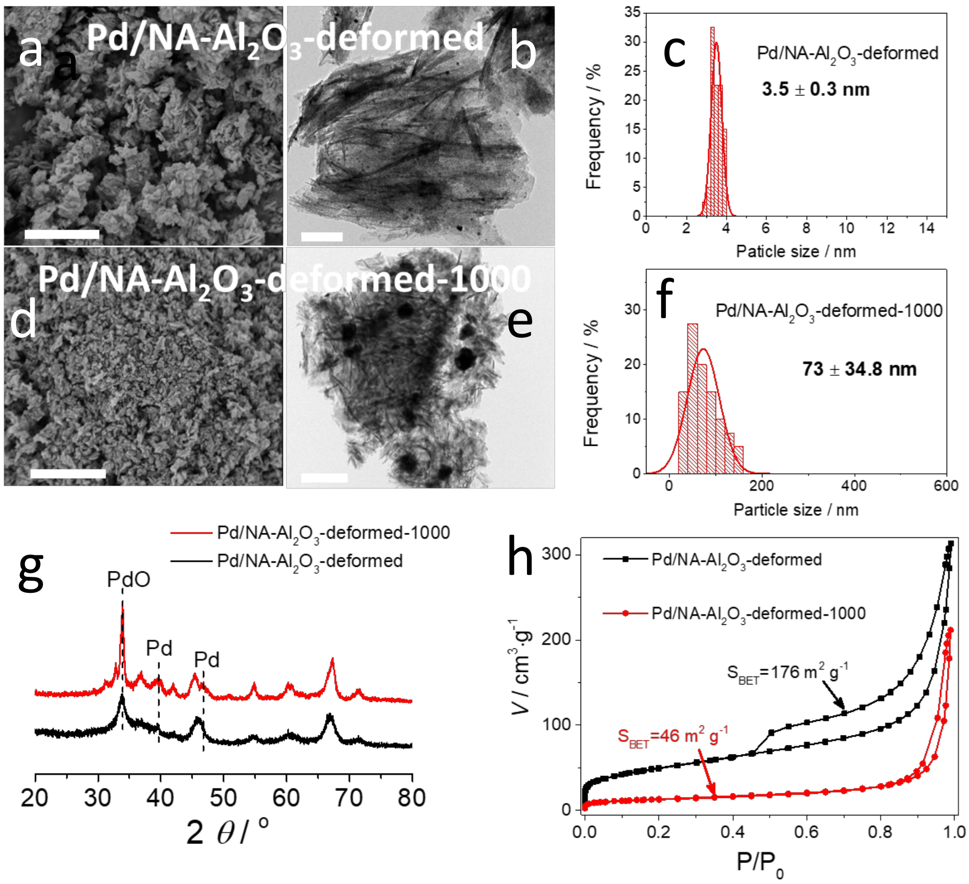

Supplementary Figure 11. a SEM images of Pd/NA-Al_2O_3-deformed. **b** TEM images of Pd/NA-Al_2O_3-deformed. **c** Particle-size distribution of Pd/NA-Al_2O_3-deformed. **d** SEM images of Pd/NA-Al_2O_3-deformed-1000. **e** TEM images of Pd/NA-Al_2O_3-deformed-1000. **f** Particle-size distribution of Pd/NA-Al_2O_3-deformed-1000. **g** XRD patterns of Pd/NA-Al_2O_3-deformed and Pd/NA-Al_2O_3-deformed-1000. **h** N_2-sorption isotherms Pd/NA-Al_2O_3-deformed and Pd/NA-Al_2O_3-deformed-1000. The scale bar in (**a**), (**b**), (**d**) and (**e**) corresponds to 2 μm, 100 nm, 2 μm and 200 nm, respectively.

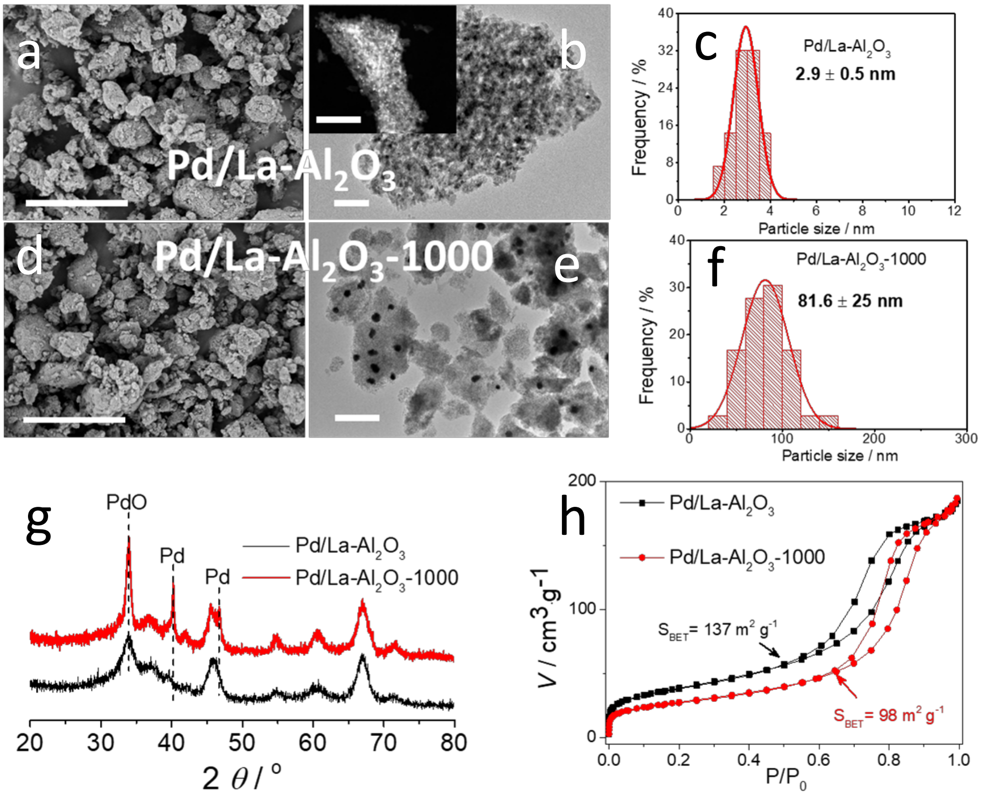

Supplementary Figure 12. **a** SEM images of Pd/La-Al_2O_3. **b** TEM images of Pd/La-Al_2O_3. **c** Particle-size distribution of Pd/La-Al_2O_3. **d** SEM images of Pd/La-Al_2O_3-1000. **e** TEM images of Pd/La-Al_2O_3-1000. **f** Particle-size distribution of Pd/La-Al_2O_3-1000. **g** XRD patterns of Pd/La-Al_2O_3 and Pd/La-Al_2O_3-1000. **h** N_2-sorption isotherms Pd/La-Al_2O_3 and Pd/La-Al_2O_3-1000. The scale bar in (**a**), (**b**), (**d**) and (**e**) corresponds to 5 µm, 50 nm, 5 µm and 500 nm, respectively. Inset in (**b**) is HAADF-STEM images of Pd/La-Al_2O_3 and the scale bar corresponds to 100 nm.

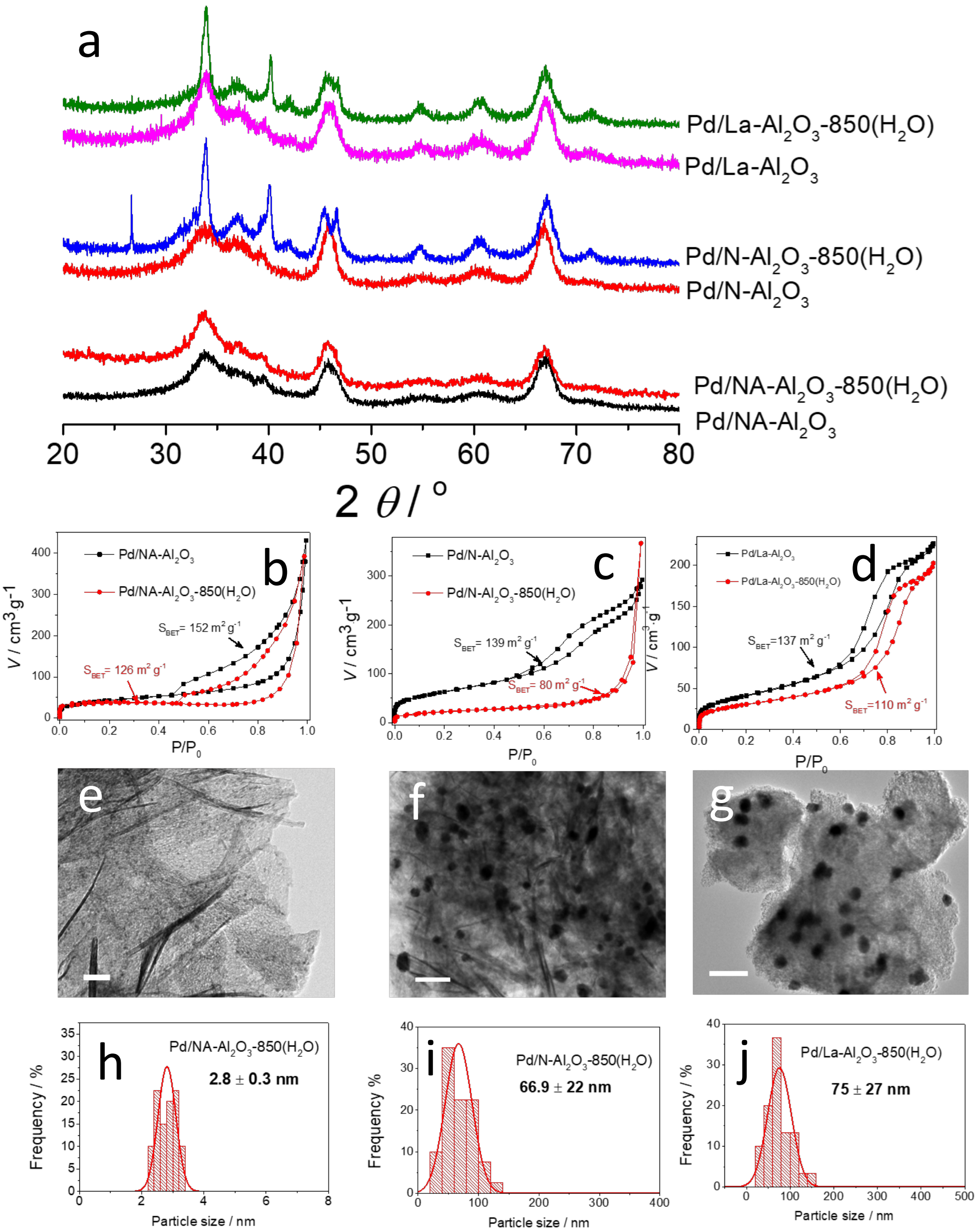

Supplementary Figure 13. a XRD patterns of Pd/NA-Al_2O_3, Pd/N-Al_2O_3 and Pd/La-Al_2O_3 before and after 850 °C-annealing in wet air (10 vol % H_2O, 20 vol % O_2 and 70 vol % N_2). **b-d** N_2-sorption isotherms of Pd/NA-Al_2O_3, Pd/N-Al_2O_3 and Pd/La-Al_2O_3 before and after 850 °C-annealing in wet air. **e-g** TEM images of Pd/NA-Al_2O_3, Pd/N-Al_2O_3 and Pd/La-Al_2O_3 before and after 850 °C-annealing in wet air. **h-j** Particle-size distribution of Pd/NA-Al_2O_3, Pd/N-Al_2O_3 and Pd/La-Al_2O_3 before and after 850 °C-annealing in wet air. The scale bar in (**e**), (**f**) and (**g**) corresponds to 50 nm, 200 nm and 200 nm, respectively.

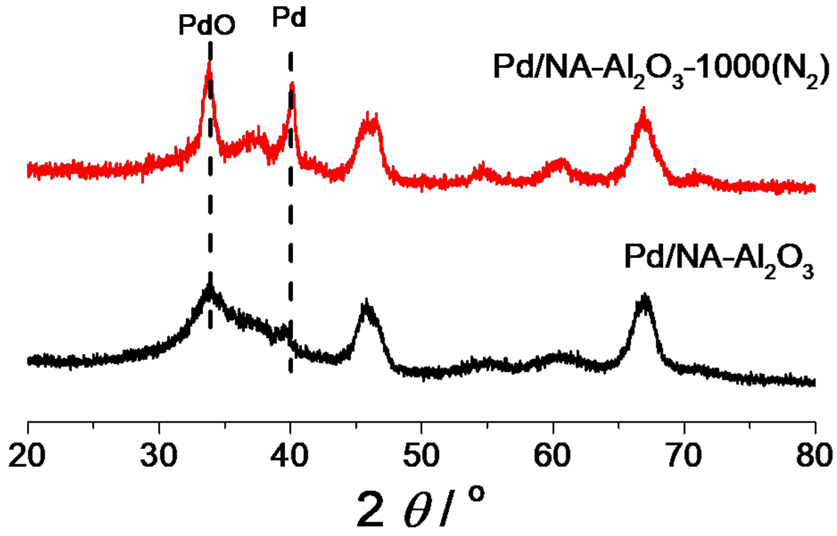

Supplementary Figure 14. XRD patterns of Pd/NA-Al_2O_3 heated in air at 1000 °C and then cooled to room temperature in N_2.

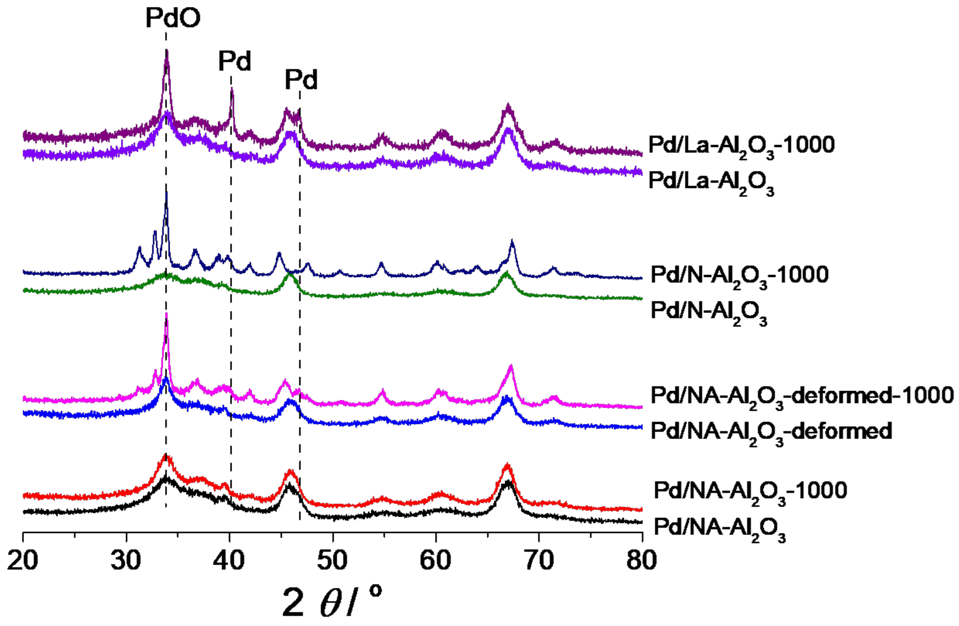

Supplementary Figure 15. XRD patterns of Pd/NA-Al_2O_3, Pd/NA-Al_2O_3-deformed, Pd/N-Al_2O_3 and Pd/La-Al_2O_3 before and after 1000 °C-annealing.

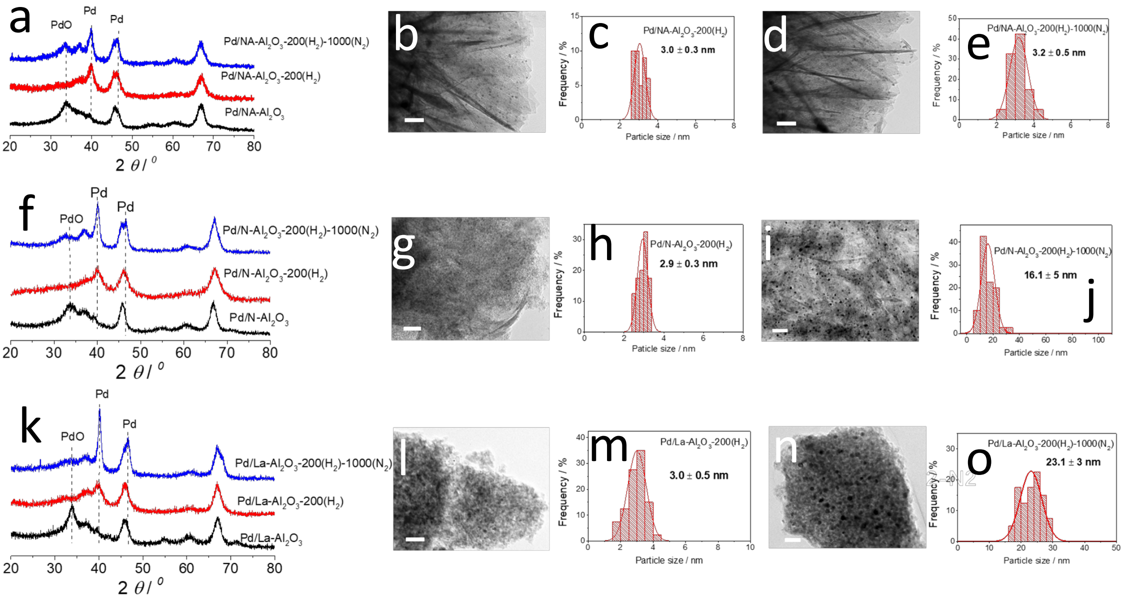

Supplementary Figure 16. a XRD patterns of Pd/NA-Al_2O_3-200 (H_2) and Pd/NA-Al_2O_3-200 (H_2)-1000 (N_2). **b** TEM images of Pd/NA-Al_2O_3-200 (H_2). **c** Particle-size distribution of Pd/NA-Al_2O_3-200 (H_2). **d** TEM images of Pd/NA-Al_2O_3-200 (H_2)-1000 (N_2). **e** Particle-size distribution of Pd/NA-Al_2O_3-200 (H_2)-1000 (N_2). **f** XRD patterns of Pd/N-Al_2O_3-200 (H_2) and Pd/N-Al_2O_3-200 (H_2)-1000 (N_2). **g** TEM images of Pd/N-Al_2O_3-200 (H_2). **h** Particle-size distribution of Pd/N-Al_2O_3-200 (H_2). **i** TEM images of Pd/N-Al_2O_3-200 (H_2)-1000 (N_2). **j** Particle-size distribution of Pd/N-Al_2O_3-200 (H_2)-1000 (N_2). **k** XRD patterns of Pd/La-Al_2O_3-200 (H_2) and Pd/La-Al_2O_3-200 (H_2)-1000 (N_2). **l** TEM images of Pd/La-Al_2O_3-200 (H_2). **m** Particle-size distribution of Pd/La-Al_2O_3-200 (H_2). **n** TEM images of Pd/La-Al_2O_3-200 (H_2)-1000 (N_2). **o** Particle-size distribution of Pd/La-Al_2O_3-200 (H_2)-1000 (N_2). The scale bar in (**b**), (**d**), (**g**), (**i**), (**l**) and (**n**) corresponds to 50, 50, 100, 100, 50 and 50 nm, respectively. Pd/NA-Al_2O_3-200 (H_2) was synthesized by reducing Pd/NA-Al_2O_3 at 200°C in 10 vol% H_2-90 vol%Ar for 30 min; Pd/NA-Al_2O_3-200 (H_2)-1000 (N_2) was synthesized by annealing of Pd/NA-Al_2O_3-200 (H_2) at 1000 °C in N_2 for 3h.

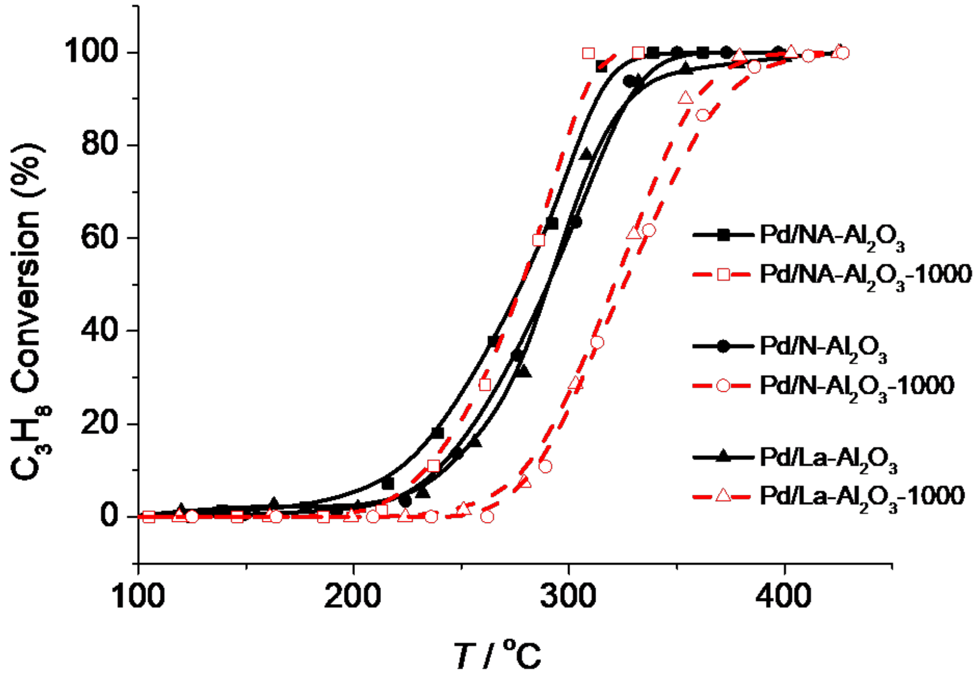

Supplementary Figure 17. Catalytic combustion of propane on Pd/Al_2O_3 nanocatalysts.

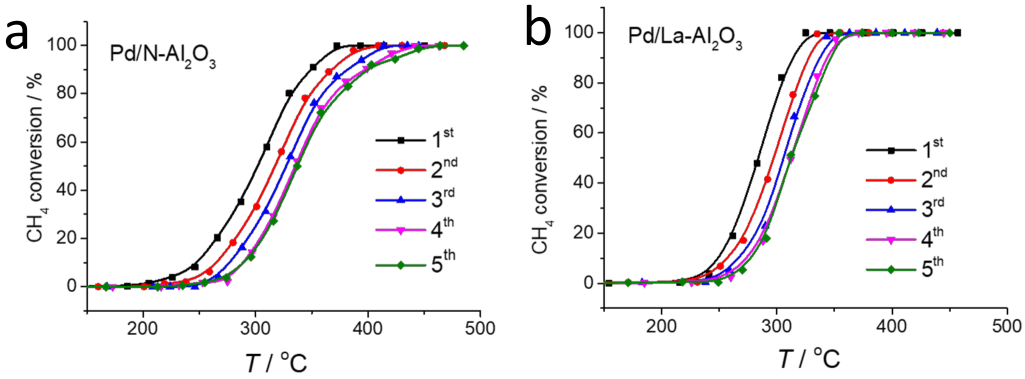

Supplementary Figure 18. Repeating ignition-extinction cycles of methane conversion on Pd/N-Al_2O_3 (**a**) and Pd/La-Al_2O_3 (**b**).

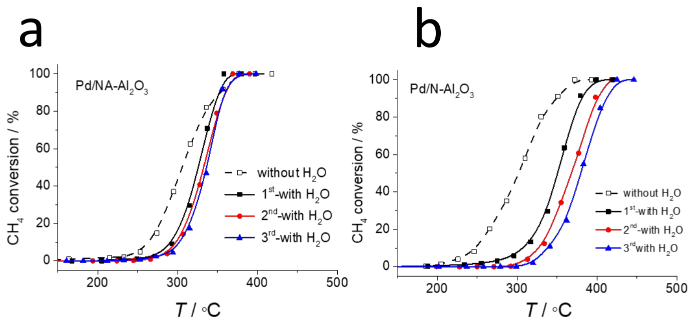

Supplementary Figure 19. Repeating ignition-extinction cycles of methane conversion on Pd/NA-Al_2O_3 (**a**), Pd/N-Al_2O_3 (**b**) and Pd/La-Al_2O_3 (**c**) under the feed gas consistent of 1 vol% CH_4, 20 vol% O_2, 10 vol% H_2O and Ar.

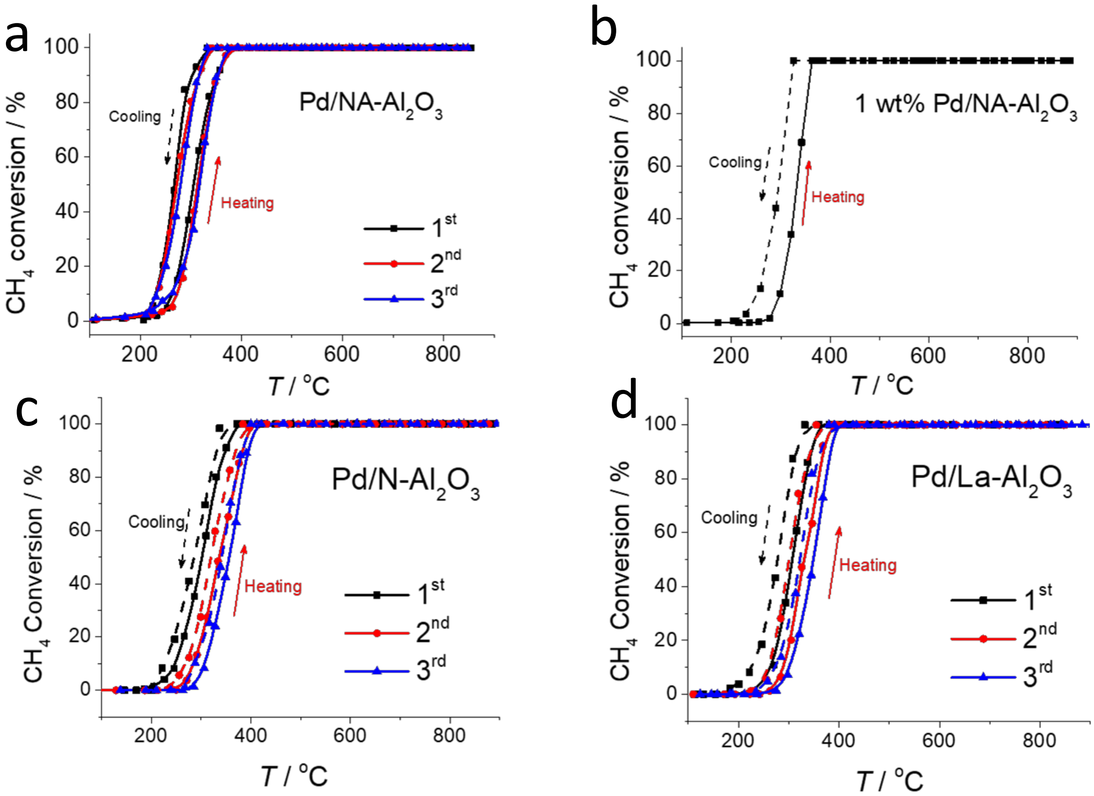

Supplementary Figure 20. Repeating light-off curves for Pd/NA-Al_2O_3 (**a** and **b**), Pd/N-Al_2O_3 (**c**) and Pd/La-Al_2O_3 (**d**).

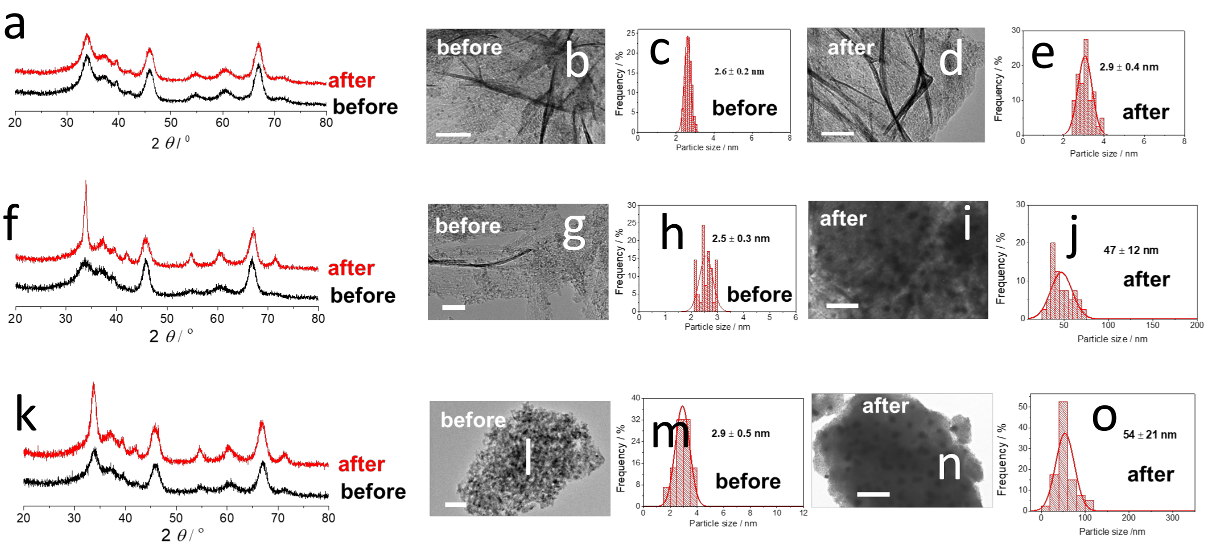

Supplementary Figure 21. Characterizations of Pd/Al_2O_3 nanocatalysts before and after long-term operation of methane combustion at 300 °C-800 °C-300 °C. **a** XRD patterns of Pd/NA-Al_2O_3 before and after reaction. **b** TEM images of Pd/NA-Al_2O_3 before reaction. **c** Particle-size distribution of Pd/NA-Al_2O_3 before reaction. **d** TEM images of Pd/NA-Al_2O_3 after reaction. **e** Particle-size distribution of Pd/NA-Al_2O_3 after reaction. **f** XRD patterns of Pd/N-Al_2O_3 before and after reaction. **g** TEM images of Pd/N-Al_2O_3 before reaction. **h** Particle-size distribution of Pd/N-Al_2O_3 before reaction. **i** TEM images of Pd/N-Al_2O_3 after reaction. **j** Particle-size distribution of Pd/N-Al_2O_3 after reaction. **k** XRD patterns of Pd/La-Al_2O_3 before and after reaction. **l** TEM images of Pd/La-Al_2O_3 before reaction. **m** Particle-size distribution of Pd/La-Al_2O_3 before reaction. **n** TEM images of Pd/La-Al_2O_3 after reaction. **o** Particle-size distribution of Pd/La-Al_2O_3 after reaction. The scale bar in (**b**), (**d**), (**g**), (**i**), (**l**) and (**n**) corresponds to 50, 50, 50, 200, 50 and 200 nm, respectively.

Supplementary Table 1 Pd dispersion of Pd/Al_2O_3 nanocatalysts

	Sample	Pd dispersion / %
1	Pd/NA-Al_2O_3	20.1
2	Pd/NA-Al_2O_3-1000	18.9
3	Pd/N-Al_2O_3	20.1
4	Pd/N-Al_2O_3-1000	7.8
5	Pd/La-Al_2O_3	21.0
6	Pd/La-Al_2O_3-1000	8.4
