# Supplementary material for: Taming the stability of Pd active phases through a compartmentalizing strategy toward nanostructured catalyst supports
Source: Nat Commun. 2019 Apr 8;10:1611. doi: 10.1038/s41467-019-09662-4 (PMC6453908; doi:10.1038/s41467-019-09662-4)
Supplement: Supplementary file 2 — Peer Review [file 41467_2019_9662_MOESM2_ESM.pdf]

Reviewers' comments:

Reviewer #1 (Remarks to the Author):

The authors present an approach to improve the thermal stability of Pd nanocatalysts for methane oxidation. The strategy involves the use of sheets of alumina to anchor the Pd metal particles. The strategy is clearly successful in preventing the nanosheets from fusing and causing a loss of surface area. The stabilization of Pd after heating to 1000 C in air is interesting and worthy of publication. But the manuscript has many technical flaws in the analysis and interpretation of the characterization data and the mechanism of Pd stabilization. These should be addressed before the manuscript is suitable for publication.

First, it would be good to compare the Pd/NA-alumina with suitable benchmarks, against which the improvement in performance was compared. For example, if one were to examine the performance of Pd on thermally stable alumina, it would be more convincing to see that the synthetic strategy developed by the authors is really beneficial. A suitable benchmark of a thermally stable alumina is missing. The comparison to the nanosheets or to commercial alumina that transforms into alpha alumina, does not provide relevant benchmarks.

The authors also confuse the stabilization of PdO and Pd metal. At 1000 C the PdO will transform into metallic Pd and this is where Pd sintering is seen. On the other hand, PdO is relatively stable and sinter resistant at moderate temperatures, under oxidizing conditions. Hence, the comparisons of stability for these two cases need to be made separately, and in each case comparisons should be to a thermally stable alumina support, such as delta alumina or La stabilized alumina.

The author's mechanism for stabilization assumes that the nanosheets expose (100) surfaces. This is never demonstrated convincingly. For example, Fig. 3 shows (100) planes of alumina. First, this indexing is incorrect, since the (100) reflection is forbidden in the alumina structure, this must be the (200) lattice fringe. But most important, the TEM looks through the sample, so these planes are actually perpendicular to the surface being studied. Hence, these images do not prove that the nanosheets actually expose the (100) surfaces. In fact, when I examined the image in Supplementary Fig. 3, I think it is a [110] zone, suggesting that it is the (110) surfaces of alumina that are exposed, and this would make perfect sense since that is the preferred surface of alumina. All of this casts doubt on the central hypothesis of the authors.

On line 182, page 9 they present an argument for why the Pd is stabilized on the NA-alumina. They show a crystal of PdO and alumina and argue there is epitaxy. But I do not see the epitaxial relationship spelled out, which involves stating the plane that is common to the two structures and a direction that is common to the two structures. Furthermore, the argument on line 190 is incorrect. The 2.04 A spacing is very common for a square planar complex of Pd, which can be found on commercial alumina surfaces. This was reported by Peterson et al., 2014 (DOI: 10.1038/ncomms5885) on a commercial alumina and confirmed by EXAFS. To argue that this form of single atom Pd is not seen on commercial alumina is not correct.

While the authors show TEM images of the Pd/N-alumina and Pd/C-alumina after 1000 C treatment in air, they do not show images of Pd/NA-alumina after such treatment. I would like to see such a comparison to clearly see the improvement achieved with the NA alumina. What they show instead is how the Pd/NA-catalyst looks after long term reaction at 330 C. But then, they do not show how the other catalysts would look after this treatment.

A particle size is given for the Pd/NA-Al2O3 sample, but not the other samples. This is needed for all the samples (before, after ageing, and even after reaction). This could be shown clearly in a particle size distribution figure (PSD). Also, the BET surface areas need to be reported.

The calculated gas hour space velocity (GHSV) appears to be incorrect. Using 200mg, with a 50ml/min flow rate would give you 15,000 ml/g-h, not 30,000 ml/g-h.

A publication Sintering Resistant Nanoparticles in Wide-Mouthed Compartments for Sustained Catalytic Performance by Jia Liu et al (DOI: 10.1038/srep41773) showed similar structures made using SiO2 that prevented Pt sintering. The authors need to demonstrate the novelty of their approach with respect to this work.

In summary, the work is potentially interesting since the NA-alumina is able to stabilize the Pd after 1000 C treatment. Generally Pd sinters under such conditions. But the comparisons they present are not satisfactory. They need to show how the Pd would perform with some relevant benchmarks, such as thermally stable aluminas, both at high temperatures and during reaction.

Reviewer #2 (Remarks to the Author):

I have mixed feelings on this paper. The alumina that the authors have prepared may have interesting properties but the authors have not proven it to me. The mere fact one can get pretty TEM images should not be the basis for publishing in a high-impact journal. The basic concept of isolating metal particles to avoid sintering is practiced on commercial materials (See Johnson Matthey Technol. Rev., 2016, 60, http://dx.doi.org/10.1595/205651316X691960, Figures 1 & 2.) but the material that the authors prepared may have novel properties. They have not proven it. My specific comments are as follows:

1) I regard it as essential that the authors report BET surface areas for their materials. Improved stability is the primary advantage that the authors claim; however, without knowing the surface areas as a function of treatment conditions, it is not possible to compare what they have to materials in the literature. The fact that another alumina that they prepared collapsed at 1000 °C does not mean other aluminas would collapse.

2) If the authors really want to claim that their materials have useful properties for methane oxidation, they need to heat their samples in wet air. Exhaust compositions are >10% water and moisture has a pretty strong effect, not only on methane-oxidation rates but on the sintering properties of alumina.

3) The XPS measurements in this paper are completely meaningless and should be removed. It is not clear how demonstrating the presence of PdO after heating to 1000 °C (and presumably cooling back to room temperature in air) really tells anything. To demonstrate that the PdO phase is stabilized, the methane-oxidation rates should have been carried up to high temperatures (for example, see Figure 5 of A. Toso, et al., Appl. Catal. B, 230 (2018) 237). If the PdO phase is stabilized, one might expect to see removal of the hysteresis curve when methane oxidation is ramped to 1000 °C, then ramped back to lower temperatures.

4) The authors write: "After a 50 h reaction, the methane conversion ratio obtained on Pd/N-Al2O3 dropped from 80.4% 225 to 75.1%, whereas the ratio obtained on Pd/NA-Al2O3 remained at ~80.3% without any change." It may be a minor point but it is meaningless to measure “rates” at 70 to 80% conversion. More importantly, their catalysts do not appear to be all that special (Figure 4). Frankly, I am surprised that cycling to 500 °C has an effect on any catalyst, which suggests the materials they are comparing their special catalyst to are pretty awful. Even so, the difference between their "special"

material and the base material are not all that different. There does not seem to be anything special about their catalyst.

Reviewer #3 (Remarks to the Author):

This is an interesting paper reporting highly stable gamma-Al_2O_3 support having Rose-like morphology. The authors demonstrated excellent stability of this material under the oxidation of methane, and the high thermal stability was attributed to three-dimensional configurations of Al_2O_3 nanosheets and abundant Al^{3+} sites for stabilization of Pd oxide particles. This paper is basically acceptable for publication in Nature Communication, however, the following information is essential.

1. Indication of these parameters of the catalyst before and after the aging is essential, surface area and Pd dispersion.

2. Methane oxidation is practically carried out in the presence of water vapor (~10%). Indicate the stability of this catalyst under the hydrothermal conditions.

Responses to the comments:

We appreciate the comments from the reviewers, and have made corrections based on their suggestions. Point-by-point responses to the reviewer comments are listed below. In addition, corrections to the manuscript are highlighted in yellow so you can easily determine the changes that were made.

Reviewer: 1

The authors present an approach to improve the thermal stability of Pd nanocatalysts for methane oxidation. The strategy involves the use of sheets of alumina to anchor the Pd metal particles. The strategy is clearly successful in preventing the nanosheets from fusing and causing a loss of surface area. The stabilization of Pd after heating to 1000 C in air is interesting and worthy of publication. But the manuscript has many technical flaws in the analysis and interpretation of the characterization data and the mechanism of Pd stabilization. These should be addressed before the manuscript is suitable for publication.
Response: Thanks very much for your professional comments. We have addressed these issues in the revised manuscript.

1. First, it would be good to compare the Pd/NA-alumina with suitable benchmarks, against which the improvement in performance was compared. For example, if one were to examine the performance of Pd on thermally stable alumina, it would be more convincing to see that the synthetic strategy developed by the authors is really beneficial. A suitable benchmark of a thermally stable alumina is missing. The comparison to the nanosheets or to commercial alumina that transforms into alpha alumina, does not provide relevant benchmarks.
Response: Thanks, it is a very good suggestion to choose a thermally stable alumina as the benchmarks to exclude the influence of phase transformation of alumina on stabilization of Pd active phase. In this study, commercial γ-phase La-doped Al_2O_3 was selected as the host materials for Pd active phases loading, owing to their excellent thermal stability that can undergo 1000 ^oC annealing treatment without phase change (Figure R 1a). In addition, to better demonstrate the advantages of our compartmentalizing strategy in stabilization of γ-Al_2O_3 from phase transformation, NA-Al_2O_3 with seriously structure deformation (deformed by mechanical grinding, Figure R 1b) was also selected for Pd active phases loading. Detailed characterization of these materials, such as XRD, SEM and N_2-sorption analysis, please see Supplementary Fig. 5 & Fig. 8 in the revised manuscript.

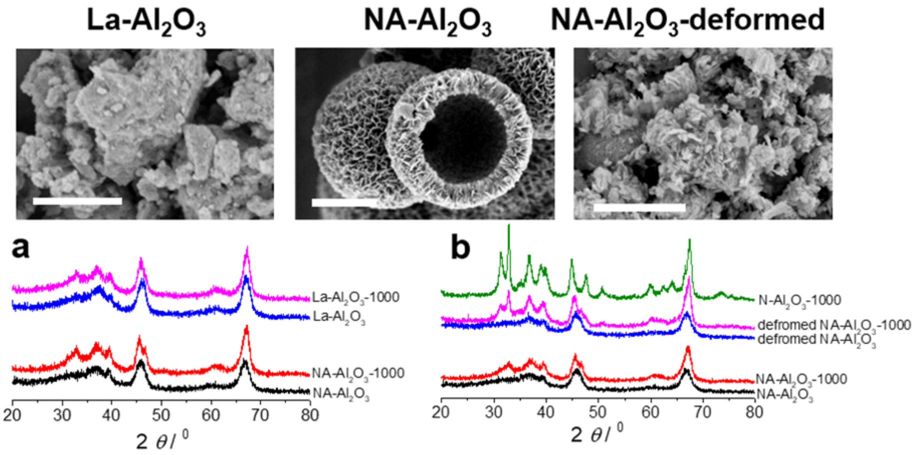

Figure R1. XRD patterns of La-Al_2O_3, NA-Al_2O_3 and NA-Al_2O_3-deformed supports before and after 1000 ^oC-annealing.

2. The authors also confuse the stabilization of PdO and Pd metal. <u>**(a)** At 1000 C the PdO will transform into metallic Pd and this is where Pd sintering is seen.</u> On the other hand, PdO is relatively stable and sinter resistant at moderate temperatures, under oxidizing conditions. Hence, <u>**(b)** the comparisons of stability for these two cases need to be made separately, and in each case comparisons should be to a thermally stable alumina support, such as delta alumina or La stabilized alumina.</u>

Response: (a) Yes, PdO will transform into metallic Pd when being subjected to 1000 ^oC-annealing in air, which is clearly identified by the arising of metallic Pd reflection at 40.1^o on the sintering samples, including Pd/N-Al_2O_3-1000, Pd/La-Al_2O_3-1000 and Pd/NA-Al_2O_3-deformed-1000 (see Fig. R2a or Supplementary Fig. 15). To better demonstrate this decomposition reaction will still happen to PdO supported on NA-Al_2O_3, Pd/NA-Al_2O_3 was annealed in air at 1000 ^oC for 3h and then cooled down to room temperature in N_2 (denoted as Pd/NA-Al_2O_3-1000(N_2)). As shown in Figure R2b (or Supplementary Fig. 14), PdO has been partially transformed into metallic Pd when cooled down in N_2. But why we only get PdO phase in Pd/NA-Al_2O_3-1000? The reason should be mainly attributed to that Pd active phases can be well stabilized in ultrasmall size by NA-Al_2O_3 at 1000 ^oC, which facilitates the complete reoxidization of metallic Pd back to PdO phase by air during the cooling process. We added these data with corresponding discussion to the revised manuscript.

(b) As suggested, we compared the stability of PdO and metallic Pd nanoparticles on thermally stable alumina (NA-Al_2O_3 and La-Al_2O_3) separately. In Figure R2c (or Supplementary Fig. 9 & Fig. 12), ultrafine PdO nanoparticles can be well stabilized by NA-Al_2O_3 after being subjected to 1000 ^oC-annealing in air, whereas an evident sintering is observed for PdO supported on La-Al_2O_3. A similar finding is discovered for the stabilization of metallic Pd nanoparticles (Figure R2d or Supplementary Fig. 16). Hence, NA-Al_2O_3 is a promising host material that can stabilize Pd active species in either oxidative phase or metallic phase at elevated temperatures. We added these data with corresponding discussion to the revised manuscript. Thanks very much for your helpful suggestions.

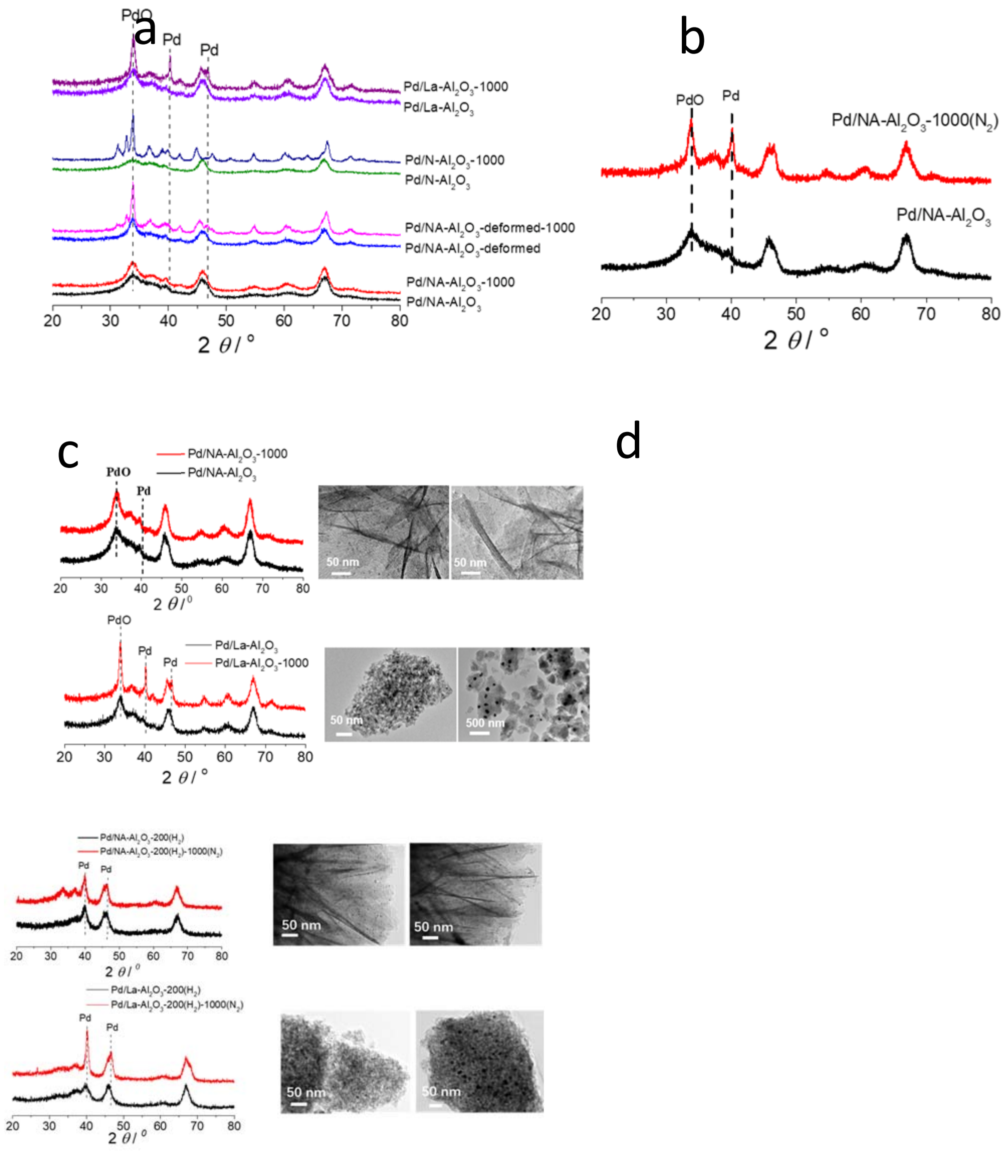

Figure R2. (**a**, **b**) XRD patterns of Pd/Al_2O_3 before and after 1000 °C-annealing; Thermal behavior of PdO (**c**) and metallic Pd (**d**) supported on Al_2O_3.

3. The author's mechanism for stabilization assumes that the nanosheets expose (100) surfaces. This is never demonstrated convincingly. For example, Fig. 3 shows (100) planes of alumina. First, this indexing is incorrect, since the (100) reflection is forbidden in the alumina structure, this must be the (200) lattice fringe. But most important, the TEM looks through the sample, so these planes are actually perpendicular to the surface being studied. Hence, these images do not prove that the nanosheets actually expose the (100) surfaces. In fact, when I examined the image in Supplementary Fig. 3, I think it is a [110] zone, suggesting that it is the (110) surfaces of alumina that are exposed, and this would make perfect sense since that is the preferred surface of alumina. All of this casts doubt on the central hypothesis of the authors.

Response: Yes, the reviewer is right. Thanks very much for his/her professional and helpful comments. The preferred surfaces of NA-Al_2O_3 are (220) facets. To demonstrate this, we have aligned the nanosheets to edge-on conditions, e.g. [112] zone axis of γ-Al_2O_3 to give direct proof of the terminal planes of NA-Al_2O_3 sheets (see Fig. R3a in the next page of this letter or Fig. 1d). The lattice fringes of $(2\bar{2}0)$ and $(11\bar{1})$ planes were clearly resolved. As you could find, the free surface of the nanosheet, now standing edge-on, are $(2\bar{2}0)$ plane (that belongs to {220} families). A schematic diagram is also given in Fig. R3a to show the geometry of nanosheet structure of NA-Al_2O_3. Again, in Fig. R3b (or Fig. 3c), we also find Pd active phases sitting on the (220) surfaces of γ-Al_2O_3. In fact, we have aligned the surfaces of several nanosheets to edge-on conditions and we found, undoubtedly, all the surfaces are {220} planes. Therefore, it is convinced that the primary exposed surfaces of NA-Al_2O_3 are {220} surfaces.

Now, the mechanism of stabilization of PdO nanoparticles by NA-Al_2O_3 becomes much more complex. We went through a lot of literatures (e.g. Science 2009, 325, 1670; Angew. Chem. Int. Ed. 2015, 54, 13994; J. Phys. Chem. Lett. 2010, 1, 2688) that utilization of pentacoordinate Al^{3+} (Al^{3+}_{penta}) centers present on (200) facet (the (200) facet was assigned as (100) facet in their studies) to stabilize metal active phases. In their study, the metal active phases, such as PtO clusters were highly dispersed and immobilized throughout the whole commercial γ-Al_2O_3 support (Fig. R3c in the next page), rather than only at certain surface of γ-Al_2O_3. However, theoretical studies pointed out that (200) facet represents about 17% total surface area of γ-Al_2O_3 (Science 2009, 325, 1670; J. Phys. Chem. Lett. 2010, 1, 2688); but, the metal active phases were dispersed on γ-Al_2O_3 everywhere. So, the question comes. How do Al^{3+}_{penta} centers present on (200) facet interact with most of the metal active phases? This still an unknown issue remained. In our study, we also demonstrated that Al^{3+}_{penta} centers plays a critical role in stabilization of PdO on NA-Al_2O_3 (Fig. R3d). It is still unknown how PdO sitting on (220) surfaces of γ-Al_2O_3 interact with Al^{3+}_{penta} centers present on (200) surfaces. This is an important but interesting question that we are going to study in our lab. In this study, our goal is to demonstrate that compartmentalization of γ-Al_2O_3 nanosheets as hierarchical structured support can greatly improve their thermal stability toward sinter-resistant nanocatalyst preparation. Thanks again for helping us correct this mistake.

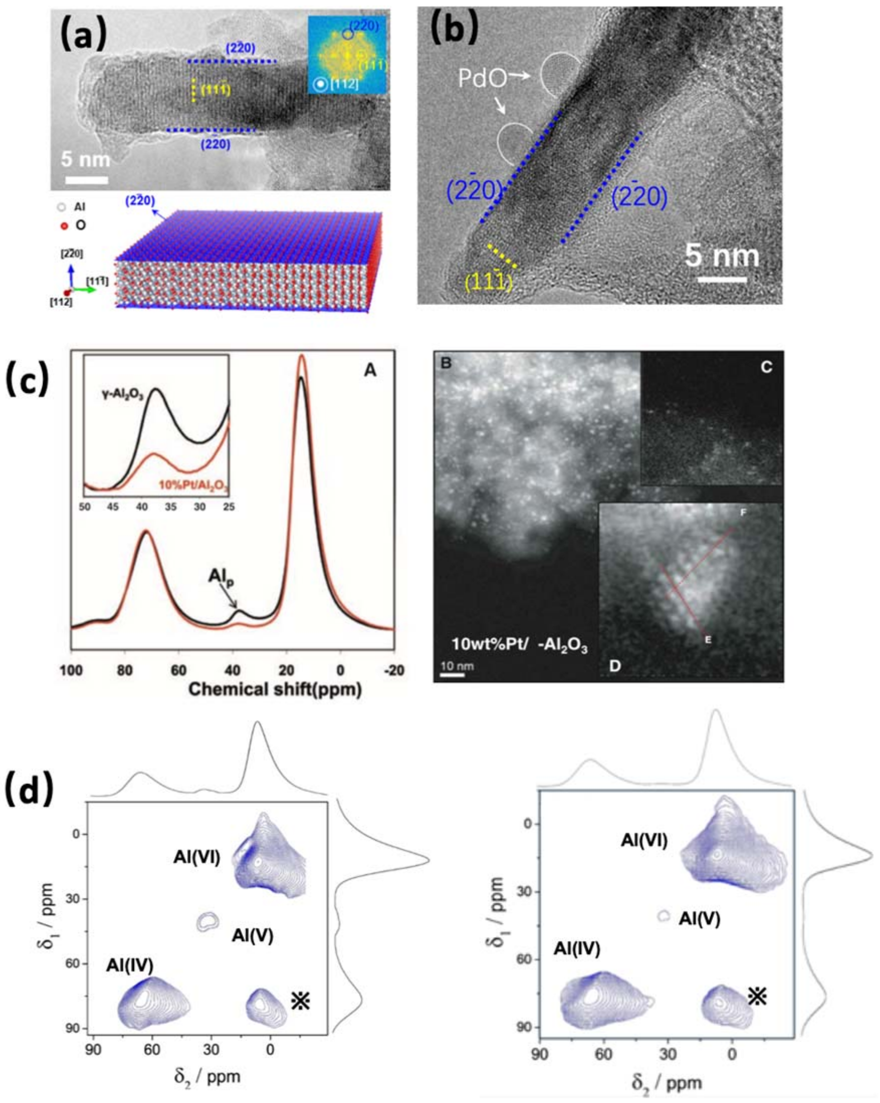

Figure R3. (**a**) TEM image and schematic diagram of nanosheet structure of NA-Al_2O_3-1000. Upper panel: HRTEM image of a nanosheet with the primary surfaces standing edge-on. The electron beam is in parallel with a [112] direction. The inset is the diffractogram from the nanosheet. It is clear that the surface of the nanosheet is terminated by a $(2\bar{2}0)$ plane. Lower Panel: A schematic diagram showing the geometry of the nanosheets. The basal plane of nanosheets are colored blue. (**b**) HRTEM image of Pd/NA-Al_2O_3-1000, where a nanosheet with its surfaces adsorbed with PdO particle. Again, the free surfaces were aligned edge-on and those planes were also identified as $\{2\bar{2}0\}$ planes. (**c**) Figures adapted from Science 2009, 325, 1670. They show that PtO nanoparticles were dispersed throughout the whole commercial γ-Al_2O_3

support with the aid of Al^{3+} (Al^{3+}_{penta}) centers present on (200) facets;(**d**) Two-dimensional ^{27}Al MQ MAS NMR spectra of NA-Al_2O_3 (left) and Pd/NA-Al_2O_3 (right).

4. On line 182, page 9 they present an argument for why the Pd is stabilized on the NA-alumina. They show a crystal of PdO and alumina and argue there is epitaxy. But I do not see the epitaxial relationship spelled out, which involves stating the plane that is common to the two structures and a direction that is common to the two structures. Furthermore, the argument on line 190 is incorrect. The 2.04 A spacing is very common for a square planar complex of Pd, which can be found on commercial alumina surfaces. This was reported by Peterson et al., 2014 (DOI: 10.1038/ncomms5885) on a commercial alumina and confirmed by EXAFS. To argue that this form of single atom Pd is not seen on commercial alumina is not correct.
Response: Yes, the reviewer is right. We have corrected these in the revised manuscript. Thanks very much for his/her professional and helpful comments.

5. While the authors show TEM images of the Pd/N-alumina and Pd/C-alumina after 1000 C treatment in air, they do not show images of Pd/NA-alumina after such treatment. I would like to see such a comparison to clearly see the improvement achieved with the NA alumina. What they show instead is how the Pd/NA-catalyst looks after long term reaction at 330 C. But then, they do not show how the other catalysts would look after this treatment.
Response: As suggested, the TEM images of Pd/NA-Al_2O_3 before and after 1000 ^{o}C-annealing in air have been added to Supplementary Fig. 9 in the revised manuscript. We also added the TEM images of Pd/La-Al_2O_3 before and after 1000 ^{o}C-annealing in air to the revised manuscript as Supplementary Fig. 12. In addition, the TEM images of Pd/N-Al_2O_3 and Pd/La-Al_2O_3 before and after methane combustion have been also added to the revised manuscript as Supplementary Fig. 21. Thanks very much for your helpful suggestions.

6. A particle size is given for the Pd/NA-Al2O3 sample, but not the other samples. This is needed for all the samples (before, after ageing, and even after reaction). This could be shown clearly in a particle size distribution figure (PSD). Also, the BET surface areas need to be reported.
Response: Thanks, the particle size distribution figures have been added to the corresponding TEM images in the revised manuscript (please see Supplementary Fig. 9-13, 16 and 21). The BET surface areas of all samples have been also added (see Supplementary Fig. 4-6 and 8-13).

7. The calculated gas hour space velocity (GHSV) appears to be incorrect. Using 200mg, with a 50ml/min flow rate would give you 15,000 ml/g-h, not 30,000 ml/g-h.
Response: Thanks, we corrected this mistake in the revised manuscript.

8. A publication Sintering Resistant Nanoparticles in Wide-Mouthed Compartments for Sustained Catalytic Performance by Jia Liu et al (DOI: 10.1038/srep41773) showed similar structures made using SiO2 that prevented Pt sintering. The authors need to demonstrate the novelty of their approach with respect to this work.

Response: In Liu's work (Figure R4a in the next page), they used the compartments of self-assembled silica nanosheets to maximize the particle-to-particle traveling distance of neighboring Pt nanoparticles to preclude the possibility of Pt sintering. In their strategy, Pt nanoparticles were placed apart from each other in wide-mouthed compartments to elongate the particle-to-particle traveling distance, and the Pt loading was optimized to be 0.07-0.35 wt% to make sure that each of the compartments contained less than one Pt nanoparticle. This is a wonderfully job, which provides a promising strategy to synthesize sintering-resistant nanocatalysts.

In this work, we demonstrate an alternative strategy for designing robust nanocatalysts through the compartmentalization of catalyst support nanostructures (Figure R4b). Different to Liu's strategy that focuses on maximizing the particle-to-particle traveling distance, the essence of our strategy lies in the formation of superstructured support through compartmentalization of 2D nanosheet materials to improve the support's thermal stability at elevated temperatures. The compartmentalized support superstructures eliminate the unique sintering channel for 2D support materials, and thus avoid the sintering and encapsulation of metal active phases by support collapsion. In addition, the metal active phases are stabilized by these superstructured supports through strong metal-support interaction, which facilitates the preparation of supported nanocatalysts with high metal loading (e.g. 5 wt%).

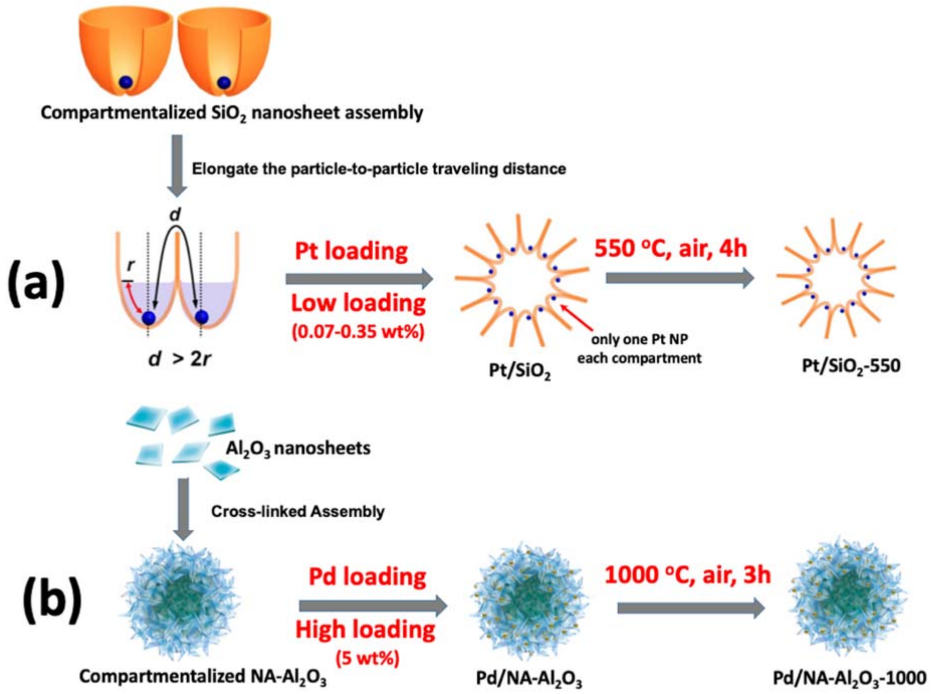

Figure R4. (**a**) Figure adapted from Jia Liu et al, DOI: 10.1038/srep41773. It shows the strategy to stabilize Pt nanoparticles on compartmentalized silica nanosheets assembly; (**b**) Illustration of stabilization of Pd active phases by NA-Al_2O_3 host materials in high-temperature oxidative conditions.

9. In summary, the work is potentially interesting since the NA-alumina is able to stabilize the Pd after 1000 C treatment. Generally, Pd sinters under such conditions. But the comparisons they present are not satisfactory. They need to show how the Pd would perform with some relevant benchmarks, such as thermally stable aluminas, both at high temperatures and during reaction.

Response: Thanks again for your professional comments and helpful suggestions. We have followed these comments and suggestions to improve our revised manuscript.

Reviewer: 2

I have mixed feelings on this paper. The alumina that the authors have prepared may have interesting properties but the authors have not proven it to me. The mere fact one can get pretty TEM images should not be the basis for publishing in a high-impact journal. The basic concept of isolating metal particles to avoid sintering is practiced on commercial materials (See Johnson Matthey Technol. Rev., 2016, 60, http://dx.doi.org/10.1595/205651316X691960, Figures 1 & 2.) but the material that the authors prepared may have novel properties. They have not proven it. My specific comments are as follows:

Response: Thanks very much for your professional comments. Different to the concept of isolating metal particles to avoid sintering, our manuscript works on improving the thermal stability of 2D nanosheet supports to prevent the sintering and encapsulation of metal active phases by support collapsion. The essence of our strategy lies in the formation of superstructured supports through compartmentalization of 2D nanosheet materials to eliminate the unique sintering channel for these 2D materials. When loaded with metal active phases, the compartmentalized superstructures may have robust thermal and mechanical stability to stabilize the supported metal active phases from sintering and encapsulation by support collapsion. This is an alternative strategy for designing robust nanocatalysts.

To better show the novel properties of synthesized NA-Al_2O_3, several control experiments have been carried out (most of them were suggested by you, we appreciate you very much). For example, La-doped γ-Al_2O_3 (La-Al_2O_3) with excellent thermal stability that can undergo 1000 ^{o}C-annealing treatment without phase change and structure collapse was selected as the benchmark for Pd active phases loading (Please see Supplementary Fig. 5 & 12); to better demonstrate the advantages of compartmentalizing strategy in stabilizing γ-Al_2O_3 nanosheets from phase transformation, NA-Al_2O_3 with seriously structure deformation (deformed by mechanical grinding, NA-Al_2O_3-deformed) was also selected as the benchmark (Supplementary Fig. 8 & 11). To evaluate the thermal stability and catalytic performance of Pd/NA-Al_2O_3 under hydrothermal conditions for practical catalytic applications, water vapor (~10 vol%) was introduced to the annealing system (Supplementary Fig. 13) and methane combustion system (Supplementary Fig. 19); to demonstrate that PdO phases are stabilized during methane oxidation, the heating/cooling ramps between 100-850 ^{o}C were collected for the study of PdO-Pd-PdO transformation (Fig. 4c and Supplementary Fig. 20). Finally, to better investigate the influence of reaction temperatures on catalytic activity, long-term operation of methane combustion at alternating reaction temperatures (e.g. 300 ^{o}C -800 ^{o}C -300 ^{o}C) was also carried out (Fig. 4d).

Thanks again for your professional comments and useful suggestions in helping us improve the revised manuscript.

1. I regard it as essential that the authors report BET surface areas for their materials. Improved stability is the primary advantage that the authors claim; however, without knowing the surface areas as a function of treatment conditions, it is not possible to compare what they have to materials in the literature. The fact that another alumina that they prepared collapsed at 1000 °C does not mean other alumin as would collapse.

Response: Thanks for your good suggestion. The BET surface areas of all samples were added to the revised manuscript (Supplementary Fig. 4-6 and 8-13). To better demonstrate the robust thermal stability of our synthesized NA-Al_2O_3, La-doped γ-Al_2O_3 (La-Al_2O_3) with excellent thermal stability that can undergo 1000 ^{o}C-annealing treatment without phase change and structure collapse was selected as the benchmark (Supplementary Fig. 5 and 12).

2. If the authors really want to claim that their materials have useful properties for methane oxidation, they need to heat their samples in wet air. Exhaust compositions are >10% water and moisture has a pretty strong effect, not only on methane-oxidation rates but on the sintering properties of alumina.

Response: Good suggestion, thanks! As suggested, two separate control experiments were carried out. Firstly, Pd/NA-Al_2O_3, Pd/N-Al_2O_3 and Pd/NA-Al_2O_3 were subjected to an annealing treatment in wet air condition (10 vol % H_2O) at 850 ^{o}C for 3 h to evaluate their thermal stability for practical catalytic applications (please see Fig. R 5a in the next page or Supplementary Fig. 13). It needs to be pointed out that 850 ^{o}C is the highest safety temperature we can reach in our lab in wet air condition. Secondly, water vapor (~10 vol%) was introduced to the reaction system to study the catalytic performance of methane oxidation under hydrothermal condition (Fig. R 5b or Supplementary Fig. 19).

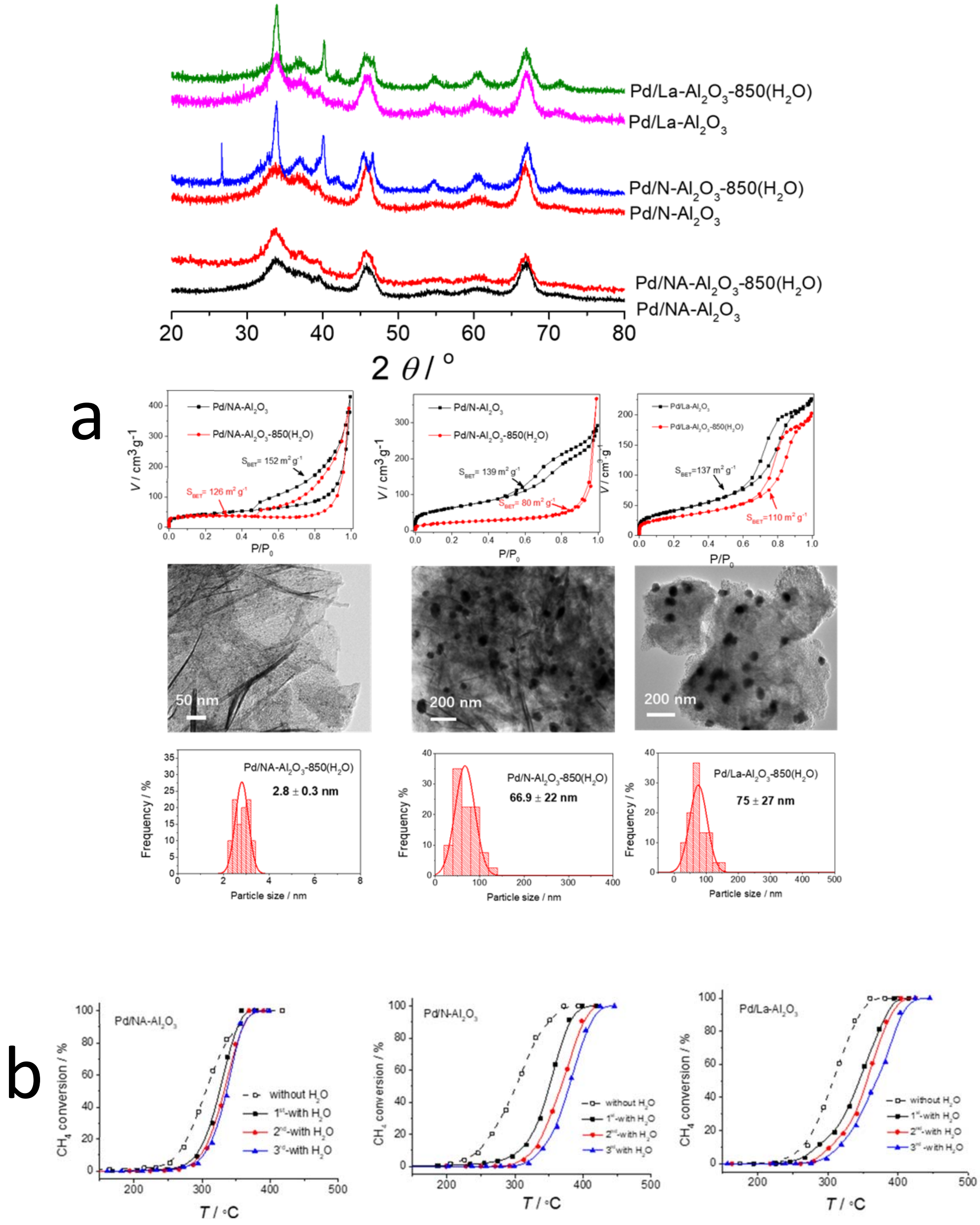

Figure R5. (a) Characterizations of Pd/NA-Al_2O_3, Pd/N-Al_2O_3 and Pd/NA-Al_2O_3 before and after being subjected to an annealing treatment in wet air condition (10 vol % H_2O) at 850 ^{O}C for 3 h; (b) Repeating ignition-extinction cycles of methane conversion on Pd/ Al_2O_3 under wet air conditions.

3. The XPS measurements in this paper are completely meaningless and should be removed. It is not clear how demonstrating the presence of PdO after heating to 1000 °C (and presumably cooling back to room temperature in air) really tells anything. To demonstrate that the PdO phase is stabilized, the methane-oxidation rates should have been carried up to high temperatures (for example, see Figure 5 of A. Toso, et al., Appl. Catal. B, 230 (2018) 237). If the PdO phase is stabilized, one might expect to see removal of the hysteresis curve when methane oxidation is ramped to 1000 °C, then ramped back to lower temperatures.

Response: As suggested, XPS spectra have been removed from the revised manuscript. To better demonstrate that PdO phase is stabilized during methane oxidation, the heating/cooling ramps carried up to high temperatures (e.g. 850 ^{o}C) were collected (Fig. R6 or Supplementary Fig. 20). The absent of typical hysteresis curve originating from PdO-Pd-PdO transformation during cooling process indicates that Pd active phases have been well stabilized in oxidative state for methane combustion. To further confirm this, a control sample with Pd loading decreased to 1 wt% (as same as the samples in Appl. Catal. B, 230 (2018) 237) was also synthesized for heating/cooling ramps measurement (Fig. R 6b). The highest safety temperature for reaction system in our lab is 850 ^{o}C, so we set up the heating/cooling ramps at temperatures between 100 ^{o}C and 850 ^{o}C. Thanks very much for your professional comments and helpful suggestions.

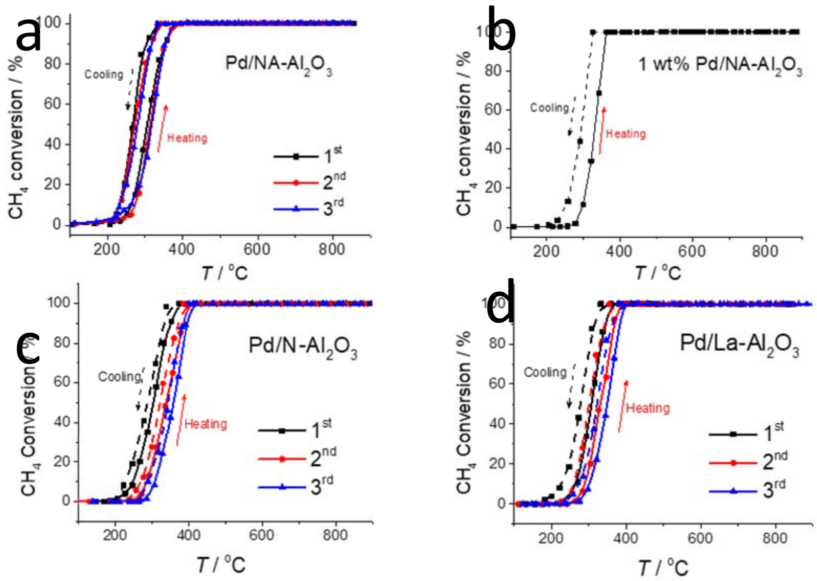

Figure R6. Repeating light-off curves for Pd/NA-Al_2O_3 (**a** and **b**), Pd/N-Al_2O_3 (**c**) and Pd/La-Al_2O_3 (**d**).

4. The authors write: "After a 50 h reaction, the methane conversion ratio obtained on Pd/N-Al2O3 dropped from 80.4% 225 to 75.1%, whereas the ratio obtained on Pd/NA-Al2O3 remained at ~80.3% without any change." It may be a minor point but it is meaningless to

measure “rates” at 70 to 80% conversion. More importantly, their catalysts do not appear to be all that special (Figure 4). Frankly, I am surprised that cycling to 500 °C has an effect on any catalyst, which suggests the materials they are comparing their special catalyst to are pretty awful. Even so, the difference between their "special" material and the base material are not all that different. There does not seem to be anything special about their catalyst.

Response: Yes, the reviewer is right. The previous catalytic activity measurements can not highlight the advantages of synthesized Pd/NA-Al_2O_3 for methane combustion. To better evaluate its catalytic performance toward methane oxidation, we re-designed the catalytic activity measurements in the revised manuscript. Firstly, water vapor (~10 vol%) was introduced to the reaction system to study the catalytic performance of methane oxidation under hydrothermal condition (Fig. R 5b in Page 9 of this letter or Supplementary Fig. 19). Secondly, to better study the influence of reaction temperatures (in particular high temperatures) on catalytic activity, heating/cooling ramps at temperatures between 100 ^{o}C and 850 ^{o}C (Fig. R6 in Page 10 of this letter or Supplementary Fig. 20) and long-term operations at alternating reaction temperatures (e.g. 300°C-800°C-300°C, Fig. R7 or Fig. 4d) were carried out for catalytic activity test. As expected, Pd/NA-Al_2O_3 demonstrated a superior catalytic performance over Pd/N-Al_2O_3 and Pd/La-Al_2O_3, such as better activity and robust catalytic durability. Thanks very much.

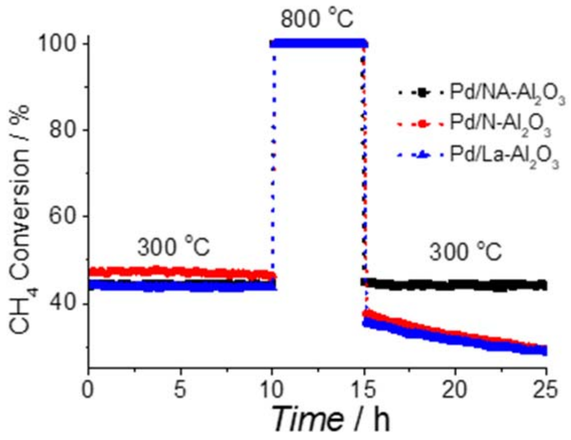

Figure R7. Long-term operation of methane oxidation at 300 ^{o}C-800 ^{o}C-300 ^{o}C on Pd/NA-Al_2O_3, Pd/N-Al_2O_3 and Pd/La-Al_2O_3.

Reviewer: 3

This is an interesting paper reporting highly stable gamma-Al2O3 support having Rose-like morphology. The authors demonstrated excellent stability of this material under the oxidation of methane, and the high thermal stability was attributed to three-dimensional configurations of Al2O3 nanosheets and abundant Al3+ sites for stabilization of Pd oxide particles. This paper is basically acceptable for publication in Nature Communication, however, the following information is essential.

Response: Thanks very much for your professional comments.

1. Indication of these parameters of the catalyst before and after the aging is essential, surface area and Pd dispersion.

Response: A good suggestion, thanks very much. The surface area and Pd dispersion of Pd/NA-Al_2O_3, Pd/N-Al_2O_3 and Pd/La-Al_2O_3 before and after the 1000 ^oC-aging have been added to the revised manuscript (please see Supplementary Fig. 9, 10, 12 and Table 1).

2. Methane oxidation is practically carried out in the presence of water vapor (~10%). Indicate the stability of this catalyst under the hydrothermal conditions.

Response: As suggested, water vapor (~10 vol%) was introduced to the reaction system to study the catalytic performance of methane oxidation under hydrothermal condition (Fig. R5b or Supplementary Fig. 19). In comparison with Pd/N-Al_2O_3 and Pd/La-Al_2O_3, Pd/NA-Al_2O_3 still demonstrated a better catalytic stability under this hydrothermal condition. Thanks very much for your professional comment.

REVIEWERS' COMMENTS:

Reviewer #1 (Remarks to the Author):

The authors have revised the manuscript, performed additional experiments (such as with 10% H_2O) and present comparisons with a benchmark La-alumina support. Additional characterization such as BET surface area, Pd dispersion and images of the samples were added.

These revisions help provide validation that the nanosheet alumina prepared in a flower-like morphology does indeed help to stabilize the Pd phase for methane oxidation reactions. The comparison between the NA alumina and the La-alumina after 1000 C aging is quite convincing. Furthermore, the preparation method appears to be simple and should be easy to replicate.

My only comment about the nomenclature is that it is perfectly correct to label the exposed surface as (110) but the lattice fringes should be indexed as (220) because the (110) reflection is forbidden for diffraction. So, the authors should make this change so readers do not get confused by seeing (220) surfaces being exposed. Also, while the edge-on TEM shows the correct orientation, the surface is very irregular. I am assuming this is because the sample is quite thick in the viewing direction, the AFM images and the low magnification views suggest that the surfaces of the flower-like alumina are quite smooth. The work does not provide any mechanistic interpretation of the enhanced sinter-resistance, but the observations presented here are worthy of publication.

Reviewer #2 (Remarks to the Author):

The authors did a very good job of addressing my concerns. I was impressed by the extra work that they performed and greatly appreciate their taking my comments and those of the other reviewers seriously.

Reviewer #3 (Remarks to the Author):

I have confirmed that the paper has been appropriately revised, and is now acceptable for the publication.

Reviewer: 1

The authors have revised the manuscript, performed additional experiments (such as with 10% H2O) and present comparisons with a benchmark La-alumina support. Additional characterization such as BET surface area, Pd dispersion and images of the samples were added.

These revisions help provide validation that the nanosheet alumina prepared in a flower-like morphology does indeed help to stabilize the Pd phase for methane oxidation reactions. The comparison between the NA alumina and the La-alumina after 1000 oC aging is quite convincing. Furthermore, the preparation method appears to be simple and should be easy to replicate.

My only comment about the nomenclature is that it is perfectly correct to label the exposed surface as (110) but the lattice fringes should be indexed as (220) because the (110) reflection is forbidden for diffraction. So, the authors should make this change so readers do not get confused by seeing (220) surfaces being exposed. Also, while the edge-on TEM shows the correct orientation, the surface is very irregular. I am assuming this is because the sample is quite thick in the viewing direction, the AFM images and the low magnification views suggest that the surfaces of the flower-like alumina are quite smooth. The work does not provide any mechanistic interpretation of the enhanced sinter-resistance, but the observations presented here are worthy of publication.

Response: Thanks for your positive comment. As suggested, we have changed the label of (220) to (110) in the revised manuscript.

Reviewer: 2

The authors did a very good job of addressing my concerns. I was impressed by the extra work that they performed and greatly appreciate their taking my comments and those of the other reviewers seriously.

Response: Thanks for your positive comment.

Reviewer: 3

I have confirmed that the paper has been appropriately revised, and is now acceptable for the publication.

Response: Thanks for your positive comment.
